# South Asian medical cohorts reveal strong founder effects and high rates of homozygosity

**DOI:** 10.1038/s41467-023-38766-1

**Published:** 2023-06-08

**Authors:** Jeffrey D. Wall, J. Fah Sathirapongsasuti, Ravi Gupta, Asif Rasheed, Radha Venkatesan, Saurabh Belsare, Ramesh Menon, Sameer Phalke, Anuradha Mittal, John Fang, Deepak Tanneeru, Manjari Deshmukh, Akshi Bassi, Jacqueline Robinson, Ruchi Chaudhary, Sakthivel Murugan, Zameer ul-Asar, Imran Saleem, Unzila Ishtiaq, Areej Fatima, Saqib Shafi Sheikh, Shahid Hameed, Mohammad Ishaq, Syed Zahed Rasheed, Fazal-ur-Rehman Memon, Anjum Jalal, Shahid Abbas, Philippe Frossard, Christian Fuchsberger, Lukas Forer, Sebastian Schoenherr, Qixin Bei, Tushar Bhangale, Jennifer Tom, Santosh Gopi Krishna Gadde, Priya B V, Naveen Kumar Naik, Minxian Wang, Pui-Yan Kwok, Amit V. Khera, B. R. Lakshmi, Adam S. Butterworth, Rajiv Chowdhury, John Danesh, Emanuele di Angelantonio, Aliya Naheed, Vinay Goyal, Rukmini M. Kandadai, Hrishikesh Kumar, Rupam Borgohain, Adreesh Mukherjee, Pettarusp M. Wadia, Ravi Yadav, Soaham Desai, Niraj Kumar, Atanu Biswas, Pramod Kumar Pal, Uday B. Muthane, Shymal K. Das, Vedam L. Ramprasad, Prashanth L. Kukkle, Somasekar Seshagiri, Sekar Kathiresan, Arkasubhra Ghosh, V. Mohan, Danish Saleheen, Eric W. Stawiski, Andrew S. Peterson

**Affiliations:** 1 Institute for Human Genetics, University of California, San Francisco, CA 94143 USA; 2 Dept of Ornithology and Mammology, California Academy of Sciences, San Francisco, CA 94118 USA; 3 MedGenome Inc., Foster City, CA 94404 USA; 4 GenomeAsia 100K Foundation, Foster City, CA 94404 USA; 5 MedGenome Labs Pvt. Ltd., Bengaluru, Karnataka 560099 India; 6 Center for Non-Communicable Disease, Karachi, Karachi City, Sindh 75300 Pakistan; 7 Madras Diabetes Research Foundation and Dr. Mohan’s Diabetes Specialties Centre, Chennai, Tamil Nadu 600086 India; 8 Thermo Fisher Scientific, Santa Clara, CA 95051 USA; 9 Punjab Institute of Cardiology, Lahore, Pakistan; 10 Karachi Institute of Heart Diseases, Karachi, Pakistan; 11 Red Crescent Institute of Cardiology, Hyderabad, Pakistan; 12 Faisalabad Institute of Cardiology, Faisalabad, Pakistan; 13 Department of Biostatistics, University of Michigan, Ann Arbor, MI 48109 USA; 14 Institute for Biomedicine, Eurac Research, Bolzano, Italy; 15 Institute of Genetic Epidemiology, Department of Genetics and Pharmacology, Medical University of Innsbruck, Innsbruck, Austria; 16 Department of Molecular Biology, Genentech, South San Francisco, CA 94080 USA; 17 Department of Human Genetics, Genentech, South San Francisco, CA 94080 USA; 18 Product Development Data Sciences, Genentech, South San Francisco, CA 94080 USA; 19 Narayana Nethralaya Foundation, Bengaluru, Karnataka 560010 India; 20 Program in Medical and Population Genetics & Cardiovascular Disease Initiative, Broad Institute of MIT and Harvard, Cambridge, MA 02142 USA; 21 Cardiovascular Research Institute and Department of Dermatology, University of California San Francisco, San Francisco, CA 94143 USA; 22 Institute of Biomedical Sciences, Academia Sinica, Taipei, Taiwan; 23 Harvard Medical School, Boston, MA 02115 USA; 24 Division of Cardiology, Department of Medicine, Brigham and Women’s Hospital, MA 02115 Boston, USA; 25 Verve Therapeutics, Cambridge, MA 02139 USA; 26 MDCRC, Royal Care Super Speciality Hospital 1/520, Neelambur, Coimbatore, Tamil Nadu 641062 India; 27 British Heart Foundation Cardiovascular Epidemiology Unit, Department of Public Health and Primary Care, University of Cambridge, Cambridge, UK; 28 National Institute for Health Research Blood and Transplant Research Unit in Donor Health and Genomics, University of Cambridge, Cambridge, UK; 29 National Institute for Health Research Cambridge Biomedical Research Centre, University of Cambridge and Cambridge University Hospitals, Cambridge, UK; 30 Health Data Research UK Cambridge, Wellcome Genome Campus and University of Cambridge, Cambridge, UK; 31 Department of Human Genetics, Wellcome Sanger Institute, Hinxton, UK; 32 Initiative for Non Communicable Diseases, Health Systems and Population Studies Division, icddr,b, Dhaka, Bangladesh; 33 All India Institute of Medical Sciences (AIIMS), New Delhi, India; 34 Medanta Hospital, New Delhi, India; 35 Medanta, The Medicity, Gurgaon, India; 36 Nizams Institute of Medical Sciences (NIMS), Hyderabad, India; 37 Institute of Neurosciences Kolkata, Kolkata, India; 38 Bangur Institute of Neurosciences and Institute of Post Graduate Medical Education and Research (IPGME&R), Kolkata, India; 39 Jaslok Hospital and Research Centre, Mumbai, India; 40 National Institute of Mental Health and Neurosciences (NIMHANS), Bengaluru, India; 41 Shree Krishna Hospital and Pramukhaswami Medical College, Bhaikaka University, Karamsad, Gujarat India; 42 All India Institute of Medical Sciences, Rishikesh, India; 43 Parkinson and Ageing Research Foundation, Bengaluru, India; 44 Manipal Hospital, Miller Road, Bengaluru, India; 45 Parkinson’s Disease and Movement Disorders Clinic, Bengaluru, India; 46 Center for Genomic Medicine, Massachusetts General Hospital, Boston, MA 02114 USA; 47 Seymour, Paul and Gloria Milstein Division of Cardiology at Columbia University, New York, NY 10032 USA; 48 Caribou Biosciences, Berkeley, CA 94710 USA; 49 Broadwing Bio, South San Francisco, CA 94080 USA

**Keywords:** Genomics, Genetics research, Genetic variation

## Abstract

The benefits of large-scale genetic studies for healthcare of the populations studied are well documented, but these genetic studies have traditionally ignored people from some parts of the world, such as South Asia. Here we describe whole genome sequence (WGS) data from 4806 individuals recruited from the healthcare delivery systems of Pakistan, India and Bangladesh, combined with WGS from 927 individuals from isolated South Asian populations. We characterize population structure in South Asia and describe a genotyping array (SARGAM) and imputation reference panel that are optimized for South Asian genomes. We find evidence for high rates of reproductive isolation, endogamy and consanguinity that vary across the subcontinent and that lead to levels of rare homozygotes that reach 100 times that seen in outbred populations. Founder effects increase the power to associate functional variants with disease processes and make South Asia a uniquely powerful place for population-scale genetic studies.

## Abstract (web summary)

South Asia is home to almost 2 billion people but is extremely underrepresented in human genetics. This study uses genomes from ~5,000 South Asians to characterize genetic variation and help facilitate future South Asian genetic studies.

## Introduction

Founder effects and population bottlenecks reduce the number of individuals from the past that contribute to present-day genetic diversity. The shifts in allele frequencies that result have contributed to many important genetic discoveries in studies of Icelandic, Ashkenazi, Finnish, Amish, and other founder or bottlenecked populations^1–4^. The historical events that have produced genetic drift in these populations are recognizable, and the genetic consequences can be effectively modeled. Studies of population structure in South Asia have described patterns of genetic drift as founder effects^5^, but there is little historical evidence that points to reductions in population size as a significant factor in producing present-day population structure. Endogamy (i.e., marriages that are restricted to a particular group or caste) is however well recognized in South Asia^6^ and can, through cultural forces for reproductive isolation, produce founder effects by reducing effective population size. In addition, consanguinity (i.e., marriages between close relatives, also called inbreeding) is common in many South Asian groups^7^ and can be thought of as an extreme form of endogamy.

Endogamy, consanguinity, population migrations, and increasing urbanization have all shaped South Asian populations in recent decades, and the degree to which the insights gained from previous studies of isolated population groups are relevant for studies involving the general patient population in the healthcare delivery system is unclear. An empirical description of population structure in patient populations is a critical first step in order to efficiently design and carry out large-scale human genetic studies in South Asia. At the same time, tools for accurate and economical genotyping, also necessary for population-scale genotyping, have been lacking for South Asians. Used together, a clear description of population structure and economical genotyping tools can unlock the tremendous potential of human genetics in South Asia for discoveries that illuminate disease processes and allow the prediction of disease risk in South Asians.

In this study, we generate and analyze high-coverage whole-genome sequence data from 4806 individuals drawn from medical cohorts in India, Pakistan, and Bangladesh, combined with 927 South Asian genomes from defined population/caste groups. We supplement standard analyses of population structure with a more detailed examination of endogamy and consanguinity, including the development of a novel method for estimating an individual’s degree of inbreeding based on the numbers and genetic lengths of autozygous tracts. We then quantify the relative effects of consanguinity and endogamy in increasing the numbers of rare homozygous variants and show how increased frequencies of rare loss-of-function (LoF) mutations in particular subgroups could be exploited for follow-up studies of particular mutations. This work highlights the unique opportunities that the genetic structure of South Asian groups provides in facilitating genotype–phenotype studies, as well as the effects of both geographic region and caste/ethnic subgroup on the underlying structure of genetic variation. Finally, we demonstrate the value of our region-specific genomic resources by constructing and validating a South Asian polygenic risk score (PRS) for coronary artery disease (CAD).

## Results

### The GAsP2 dataset

We divided samples from the healthcare delivery systems of South Asia (informally called “medical cohorts”) into three regional groups: Pakistani (PKN), South Indian (SOI), and Bengali (BNG). To allow comparisons with previous studies characterizing South Asian population structure, we combined these samples with previously published genomes^8–13^ and added newly sequenced genomes from defined South Asian population groups to create the GenomeAsia Phase 2 (GAsP2) dataset. We then used a standard pipeline for read mapping and variant calling, starting from the raw sequence data of all of the samples. After standard quality control filters and the removal of one individual from each first-degree relative pair, we obtained a set of 6442 high-coverage genomes (average 25×) for downstream analyses (Supplementary Data 1). Of these, 5734 genomes were of South Asian ancestry (5118 of which were sequenced for this study), with medical cohort sizes of 1810, 1362, and 1634, respectively, for the Pakistani, South Indian, and Bengali groups. Basic information on SNPs and allele frequencies is available from the GenomeAsia consortium website (https://www.genomeasia100k.org), while a computationally phased version of the data can be used as a reference panel for imputation using the Michigan Imputation Server (https://imputationserver.sph.umich.edu/index.html). The estimated non-reference discordance rate for duplicate samples found in both the GAsP2 and 1000 Genomes Project datasets is 1.61 × 10^−4^ (Supplementary Table 1).

### Population structure

**Fig. 1:**
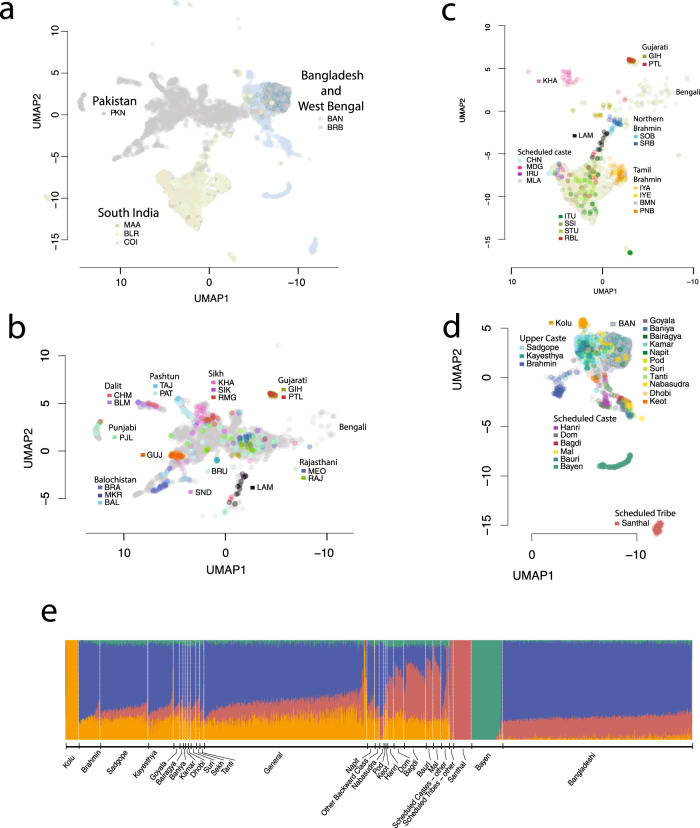
Fine-scale population structure in the healthcare delivery system reflects the geographical locations of the sample sources. UMAP was run on all samples using the first 15 principal components. **a** In the South Asian subset, samples cluster into three major groups by sample origins: Pakistan, South India, and West Bengal and Bangladesh. The *X*-axis (UMAP1) was flipped so that the similarity between the graphical position of the three populations and the map of South Asia was apparent. **b**, **c** Samples with detailed locations or self-reported group memberships are shown to segregate within Pakistan and South India clusters. Among the samples from Pakistan and South India, some segregate with recent immigrants (e.g., Bengalis and Gujaratis) and historical immigrants (e.g., Lambadas), reflecting the metropolitan nature of the recruitment centers. **d** Samples from Birbhum District, West Bengal, have detailed self-reported group membership information. Upper castes, scheduled castes, and scheduled tribes clearly segregate, reflecting the historical reproductive isolation between these groups. Bayen and Santhal are two notable population isolates. **e** ADMIXTURE analysis of samples from the Birbhum District shows four major components. Labels are self-reported group identities with “general” denoting a lack of specified identity. PKN Pakistan, BLR Bangalore, MAA Chennai, COI Coimbatore, BAN Bangladesh, BRB Birbhum District, West Bengal, LAM Lambada. For other 3-letter codes, see Supplementary Data 1.

We used standard approaches such as F_ST_, PCA^14^, Admixture^15^, and Uniform Manifold Approximation and Projection (UMAP)^16^ for qualitatively assessing population structure in our study (Fig. 1 and Supplementary Figs. 1–4). In the UMAP plot of South Asian samples, we find a clear distinction between Bengali, Pakistani, and South Indian groups that roughly mirror geography (Fig. 1a). When we further zoom in on each of these three regions, we find that UMAP can separate individuals into smaller caste and culture-based subgroups (Fig. 1b–d). In particular, since our dataset includes genomes from both medical cohorts (sampled from particular regions without regard to caste) and focused sampling of genomes from identified caste and language groups, we can sometimes reliably assign caste (Fig. 1d) or subgroup (Fig. 1b) labels to patient genomes based solely on their genetic makeup.

In Birbhum, West Bengal, we have self-reported subgroup identity (e.g., tribe, caste, and/or sub-caste) for over half of the individuals in our study that were recruited through the healthcare delivery system. Figure 1c shows that some self-reported subgroup identities form distinct genetic clusters, such as the Santhal, Bayen, and Brahmin, while others do not, like the Sadgope and Kayesthya. An admixture plot of Birbhum and Bangladeshi samples with *K* = 4 provides a similar picture (Fig. 1e), with Santhal, Bayen, and Kolu appearing to be well-defined genetic groups, while most individuals from the other groups are estimated to be admixed. The clustering of the Santhal, Bayen, and Kolu reflects increased genetic drift due to some combination of isolation, endogamy, and consanguinity. It is well established that increased drift in founder populations can enable unique opportunities for genetic research, and drift in South Asian populations support the idea^5,17^ that this will prove true here as well. Among the other population groups in our study, we note that there is a clear distinction in Admixture estimates between Bangladeshis and general caste individuals from Birbhum (who include a substantial number of Bengali Muslims). Since Partition (between India and what was then East Pakistan) happened too recently to cause systematic genetic differences, our results suggest that either our Bangladeshi or our Birbhum Muslim samples (or both) are not representative of the Muslims that were living in Bengal in the mid-twentieth century.

### Endogamy and consanguinity

**Fig. 2:**
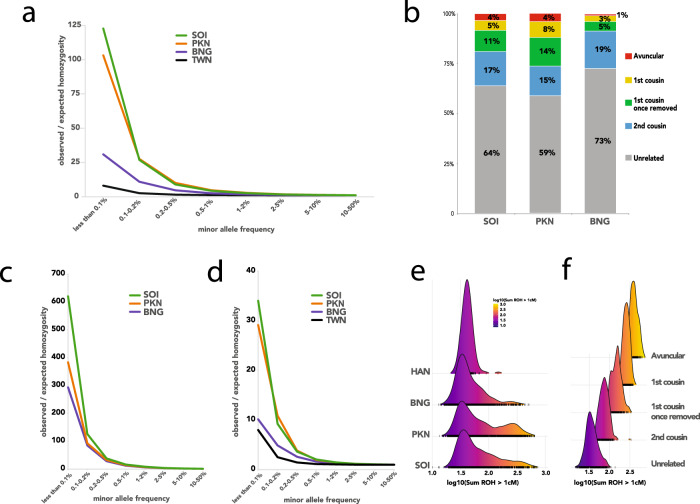
Homozygosity and inbreeding across different cohorts. **a** Observed/expected proportions of rare homozygotes, stratified by minor allele frequency and population. The expected values assume random mating. **b** Stacked bar chart showing the estimated degree of inbreeding for individuals in the South Asian medical cohorts. **c** Same as in panel **a** but for “inbred” individuals (whose parents are estimated to be third-degree relatives or more closely related) only. **d** Same as in panel **a** but for “outbred” individuals (whose parents are estimated to be sixth-degree relatives or more distantly related) only. **e** Ridgeplots showing the distribution across individuals of the total (genetic) length of the genome contained in ROHs that are at least 1 cM in length. **f** Ridgeplots showing the stratification of panel **e**’s PKN plot into groups with different estimated degrees of inbreeding.

Endogamy and consanguinity lead to an excess of homozygous genotypes over the expectations from random mating. We calculated the ratio of the observed number of rare homozygous genotypes over the expected number, binned by minor allele frequency (MAF), for South Indian (SOI), Pakistani (PKN), and Bengali (BNG) samples (Fig. 2a). For comparison with the patterns of genetic variation in an ostensibly outbred population, we also included the same results for 1442 unrelated Taiwanese (TWN) genomes from the Taiwan Biobank^18^. For all four groups, we observe an increasing excess of rare homozygotes with smaller MAF. This pattern is strongest in the South Indian and Pakistani groups, moderate in the Bengali group, and weak in the Taiwanese, and reflects the relative strength of non-random mating within each group.

Population bottlenecks that occurred in the distant past can dramatically increase the rate of homozygosity, but with tract lengths that decrease over the increasing numbers of intervening generations. Endogamy and consanguinity also both produce excess homozygosity, but in the latter, the excess homozygosity primarily occurs in long tracts or runs of homozygosity (ROH). To determine the relative effects of endogamy and consanguinity on patterns of homozygosity within our South Asian cohorts, we developed a novel method for estimating the degree of parental relatedness of an individual based on the observed numbers and lengths of long (i.e., >10 cM) ROHs. The method categorized an individual’s parents as second-degree relatives (e.g., avuncular, such as uncle and niece), third-degree relatives (e.g., first cousins), fourth-degree relatives (e.g., first cousins once removed), fifth-degree relatives (e.g., second cousins) or unrelated (i.e., less related than second cousins). Both the proportion of individuals identified as outbred and the distribution of consanguineous individuals across the remaining four categories show substantial regional variation (Fig. 2b). In particular, there appears to be less consanguinity and fewer closely related parental pairings on average among the individuals in BNG (West Bengal and Bangladesh), compared with the medical cohorts from SOI (South India) and PKN (Pakistan). This presumably reflects systematic differences in marriage practices across the different regions. Endogamy is the result of in-group mate choice rules that often use distinct patrilineal and matrilineal definitions of relatedness that do not align with the descriptions of relatedness based on autosomal inheritance. Given that cultural concepts of relatedness can differ between different endogamous groups and are oftentimes closely held, it is perhaps not surprising that self-reported consanguinity in the Birbhum samples is only modestly correlated with genetic estimates of consanguinity (Supplementary Fig. 5).

To assess whether consanguinity can by itself explain the observed excess of rare homozygotes, we stratified each regional group into “inbred” and “outbred” subgroups (where the former referred to individuals whose parents were estimated to be second- or third-degree relatives). We then tabulated the increase in rare homozygotes for each subgroup (Fig. 2c, d). We find that the inbred subgroups (Fig. 2c) have as much as 600 times higher levels of rare homozygotes above random mating expectations, 4–10-fold above the population group as a whole and 13–30 times the level seen in the corresponding outbred subgroups. Interestingly, even in the outbred South Asian subgroups (Fig. 2d) levels of homozygosity can be as much as 3.8-fold higher than in TWN (Han Chinese from Taiwan) individuals, presumably due to endogamy and the resulting enrichment in distant parental relationships for the individuals in our dataset which our methods were not able to identify.

Excess homozygosity caused by close parental relatedness is structured in very long ROH. We tabulated the total length of each individual’s genome contained in ROH longer than 1 cM, and plotted the distribution of this sum across several South Asian groups, along with HAN (Han Chinese from China) as an outbred population for comparison (Fig. 2d, e). All of the South Asian groups have a tail of individuals with more of their genomes in ROH tracts longer than 1 cM (Fig. 2e). Individuals with a greater sum of ROH tracts are correlated with those inferred to be consanguineous (Fig. 2f and Supplementary Fig. 6) consistent with the idea that recent inbreeding is correlated with the presence of additional consanguinity loops involving more distant relatives.

### Loss of function variants

**Fig. 3:**
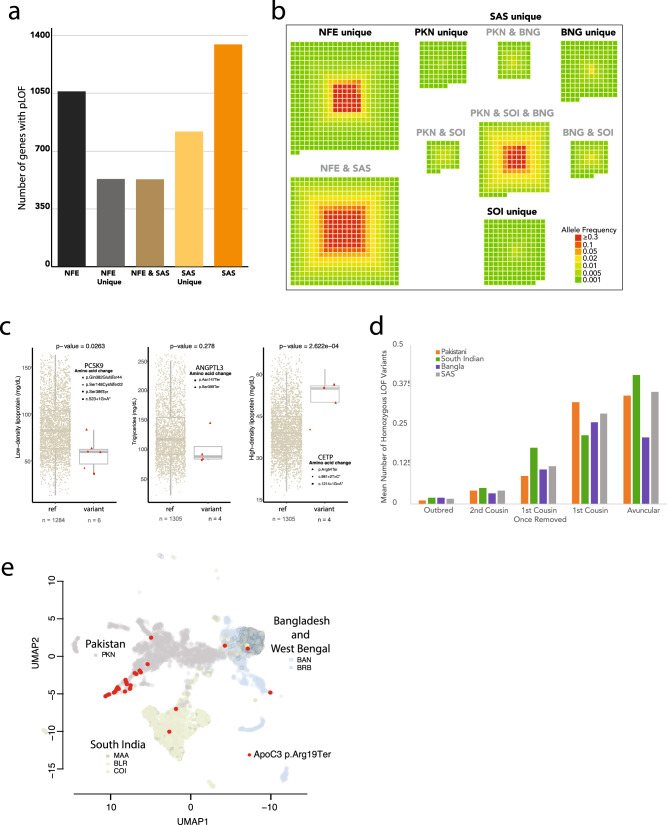
Loss of function mutations. **a** Number of high confidence loss of function genes found at a minimum of 0.1% MAF in their relative population for overall non-Finnish European (NFE), NFE and not in SAS (NFE Unique), NFE and SAS (NFE and SAS), SAS and not NFE (SAS Unique) and overall for SAS. **b** Loss of function gene space by population. Each square represents a distinct gene and is colored by its maximum AF within the relative group. Genes are separated by groups in which they are found (from top to bottom and then left to right): NFE unique, NFE and SAS, PKN unique, PKN and SOI, PKN and BNG, all of SAS (PKN and SOI and BNG), SOI unique, BNG unique, and BNG and SAS. **c** Effects of pLoF variants on blood lipid markers replicated the known biology: *PCSK9* pLoFs associated with decreased LDL, *ANGPTL3* pLoFs associated with decreased triglycerides, and *CETP* pLoF associated with increased HDL. Only samples from South India (Bangalore and Chennai) were included. *P* values were calculated using the Wilcoxon rank-sum test. Box shows median and middle 50% of the distribution; whiskers show values within 1.5 times the interquartile range from the first and third quartiles. **d** Mean number of homozygous pLoF variants per individual, stratified by population and estimated degree of inbreeding. **e** *APOC3* p.Arg19Ter alleles are found at a high frequency among Balochi and Sindhi individuals from Southern Pakistan. Three of the self-reported Balochis and Sindhis were heterozygous carriers, but a larger number of carriers without self-reported identity were mapped to the same region on the UMAP plot.

To assess the potential functional effects of the high levels of endogamy and consanguinity found within South Asia, we identified putative loss of function (pLoF) variants in our dataset (see Methods) and focused on genes containing at least one pLoF variant at frequency > 0.1% across the combined South Asian medical cohorts (i.e., SAS). For comparison, we also used an analogous analysis pipeline to identify genes containing pLoF variants in non-Finnish European (NFE) individuals from the genome aggregation database^19^ (gnomAD). There are more genes with pLoF variants with frequency >0.1% found in South Asia than in NFE, and most (~61%) of these genes are in the set of pLoF-containing genes unique to SAS (Fig. 3a). To visualize the frequencies and geographic distributions of these pLoF variants, we constructed heat maps representing genes containing pLoF variants, with warmer colors indicating higher minor allele frequencies of pLoF variants. (The heat map scale is for the minor allele frequencies in SAS and/or NFE.) Individual clusters are shown for each set of pLoF-containing genes that are unique to or shared between individual population groups (Fig. 3b). The large number of pLoF genes that are not shared across all three South Asian medical cohorts is consistent with the idea that SOI, BNG, and PKN are distinct population groups, each of which having experienced genetic drift pushing LoF and other functionally relevant alleles to higher frequencies than is seen in outbred populations. We also show the same heat maps for homozygous pLoF variants (i.e., human “knockouts”) in Supplementary Fig. 7.

pLoF mutations are widely studied because they often have phenotypic effects that can easily be tied to the function of a specific gene or pathway. We looked at three genes where LoF mutations are known to affect serum lipid levels^20–22^ and verified that individuals in our study that have pLoF variants in these genes have the expected effects on measured LDL, triglyceride, and HDL levels (Fig. 3c).

A recognized benefit of studying South Asian populations is the greater probability of identifying individuals homozygous for pLoF alleles due to the excess homozygosity caused by endogamy and consanguinity. To explicitly evaluate this potential in our dataset, we tabulated the average numbers of rare (MAF < 0.01) homozygous pLoF mutations per individual (i.e., those pLoF mutations most likely to be deleterious), stratified by estimated degree of consanguinity (Fig. 3d). As expected, increased consanguinity is associated with an increased number of these rare, likely harmful mutations, similar to previous findings (cf. Figure 1c in ref. ^23^). As the degree of consanguinity increases, these mutations are more likely to be found in long ROH caused by recent inbreeding (Supplementary Fig. 8).

The population structure within South Asia makes the region ideal for prospective studies of LoF mutations. Even rare pLoF variants might have an appreciable frequency in particular regions or caste groups, which would enable focused recruiting for follow-up functional studies. To evaluate this, we displayed the distribution of individuals containing characterized LOF alleles on the UMAP plots described previously (Fig. 3e and Supplementary Fig. 9). Characterization of ApoC3 LOF homozygotes has elucidated the physiological basis by which ApoC3 acts to regulate serum triglycerides^23^. Within South Asia, ApoC3 LOF carriers are found predominantly in Pakistani subpopulations that cluster with individuals from Balochistan and Sindh in the South of Pakistan (Figs. 1b and 3e).

### The SARGAM genotyping SNP array

**Fig. 4:**
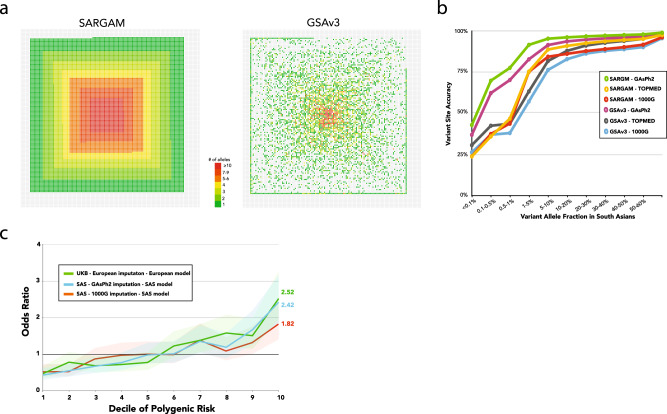
Improved genotyping of South Asian genomes. **a** Gene space plot of all protein-altering alleles that are directly genotyped using either the SARGAM or the Illumina GSA3 arrays. Protein-coding genes of the human genome are depicted as an array of 19,600 squares. Genes whose variants are genotyped are colored to indicate the number of gene-specific variants that are genotyped. **b** Accuracy of non-reference allele imputation expressed as the concordance rate and plotted versus South Asian minor allele frequency. Array genotypes were modeled by down-sampling from an independent dataset of 30× WGS data. Missing genotypes were imputed using the indicated reference panels and the variant site accuracy of non-reference alleles was calculated and graphed for variants imputed from the two indicated model array datasets. **c** Impact of imputation on polygenic risk score (PRS) calculation. PRS were calculated using imputed genotypes from a CAD case–control cohort of 2963 South Asian individuals genotyped using the Illumina GSA3 array and using a SAS PRS model^24^. The individuals were divided into 10 groups based on deciles of PRS and odds ratios were calculated from the case–control status of the individuals in each group. For comparison, a case–control cohort of white Britons, matched for age and gender with the SAS cohort, was selected from the UK Biobank dataset. PRS was calculated using a European model; point estimates of the odds ratios are displayed as solid lines for each PRS, and the corresponding 95% confidence intervals (using the empirical variance based on the case/control counts in each decile) are shown as a shaded area.

To optimize the effectiveness of future genotype-phenotype studies in South Asia, we worked with Thermo Fisher to design a custom SNP array (South Asian Research Genotyping Array for Medicine, or SARGAM) that (i) prioritizes direct genotyping of known or putative protein-altering variants present at a frequency of 0.1% or higher in SAS populations, and (ii) is optimized for imputation of variants (quantified by non-reference concordance rate) down to an SAS minor allele frequency of 0.1%. To highlight the former feature, we tabulated how many pLoF or presumed damaging mutations can be directly genotyped by the most commonly used technology at present, Illumina’s GSA3 array and by the SARGAM array (Fig. 4a, b and Supplementary Fig. 10). In Fig. 4a, b, each protein-coding gene is represented by a square in an array of 19,600 squares, with each square colored by the number of deleterious variants captured by each array at each gene. The SARGAM array directly genotypes presumed damaging mutations from the vast majority (74%, *n* = 14,713) of non-readthrough (cf. https://www.ensembl.info/2019/02/11/annotating-readthrough-transcription-in-ensembl/) protein-coding genes in Ensembl (Fig. 4a), with a mean coverage of 3.5 mutations per gene (*n* = 51,804). In contrast, the number of damaging mutations genotyped by the GSA3 array is much smaller (Fig. 4b), covering just 26% of genes (*n* = 5100) with a mean coverage of 1.9 mutations per gene (*n* = 9443). The SARGAM array therefore represents an inexpensive method for simultaneously conducting many specific genetic tests, while also allowing for standard human genetic applications (e.g., genome-wide association studies and/or PRS calculations).

Since the SARGAM array design utilized the observed patterns of linkage disequilibrium in thousands of South Asian genomes, we expected it to allow for more accurate imputation of untyped genotypes in South Asian samples. To evaluate this, we compared imputation accuracy between the simulated SARGAM and GSA3 arrays and found that both the SARGAM array and the GAsP2 reference panel contribute to higher imputation accuracy (Fig. 4b). These results reinforce the conclusion that the quality of available genomic resources is a significant factor that propagates existing disparities by limiting the feasibility and power of large-scale human genetic studies in non-European populations.

### Polygenic risk scores and the genetic architecture of complex traits

We demonstrate the clinical relevance of the improved genotyping and imputation through an application of CAD PRSs in an independent South Asian cohort (1800 cases, 1163 controls) which were genotyped on the GSA3 arrays^24^. We imputed the genotypes using the 1000 Genomes and GAsP2 panels and applied the ancestry-adjusted genome-wide PRS model from ref. ^24^. The results showed a marked improvement in the predictive power of the PRS, with an improved AUC (0.638 for GAsP2 vs. 0.595 for 1000 Genomes). The odds ratios of CAD for individuals in the top deciles (ninth–tenth) compared to those in the middle deciles (fifth and sixth) are higher in the GAsP2-based PRSs (OR_9th_ = 1.67; OR_10th_ = 2.43) as compared to the 1000 Genomes-based PRSs (OR_9th_ = 1.32; OR_10th_ = 1.83; Fig. 4c). These improved ORs are on par with those achieved for European samples with the appropriate imputation panel (HRC, UK10K, 1000 Genomes), GWAS, and PRS model (UKB OR_9th_ = 1.51; OR_10th_ = 2.52; Fig. 4c). This improved performance can be explained by the improved imputation accuracy as well as the increased number of well-imputed variants (Supplementary Table 2).

## Discussion

South Asian populations provide a rich potential for human genetic discovery that is largely unexplored. A population-scale genotyping project in South Asia will open up opportunities to explore disease genetics in ways that are impractical or infeasible in other populations. Notably, the dramatically higher rate of homozygosity that is found in parts of South Asia allows homozygous loss of function effects to be studied for many genes that cannot realistically be accessed in outbred populations such as those that predominate in Europe and East Asia^19,25^. Traditionally, homozygous gene function has been explored through family-based studies, often involving self-identified consanguineous unions. Although this will continue to be an effective way to carry out focused studies, a population-scale dataset in South Asia will facilitate the identification of appropriate families and will also open up new opportunities to consider homozygosity in population-based association analyses. At the same time, this dataset will provide the opportunity to evaluate disease associations with a novel set of functional variants, e.g., the unique and larger set of pLoF alleles with frequencies >0.1% found in South Asians as compared to Europeans. The SARGAM genotyping array and the GAsP2 imputation reference panel allow South Asian genotypes to be captured in an economical and effective manner.

Population-based genetic studies have been effectively carried out within single coordinated healthcare delivery systems such as national single-payer systems. South Asia provides a different set of challenges and a different set of possibilities. In India in particular, super-specialty hospitals, organized to deliver healthcare in a specific disease area in a way that takes advantage of the economies of scale presented by its large population base, predominate in certain markets. These hospitals can, in a disease-focused fashion, rival the scale of national general hospital systems of some countries. Thus while national biobank systems do not exist in South Asia at present that could provide a foundation for a broad cross-sectional evaluation of the genetics of disease, the scale at which patients can be recruited within specific disease areas will allow clinically relevant datasets to be constructed to a total size that is unrealistic in most parts of the world. This, paired with the unique population structure of South Asia, presents a powerful set of opportunities for genetic discovery that will improve healthy life span around the globe.

## Methods

Our research was conducted in compliance with the ethical committees associated with each of the sampling sites. Specific details are provided in Supplementary Note 1.

### Samples

We utilized a combination of genomes from previously published studies^8–13^, newly sequenced genomes from 1000 Genomes Project samples, and newly sequenced genomes from several ongoing genetic studies in South Asia. Further information on the samples is contained in Supplementary Note 1 and Supplementary Data 1. Our study contains 5118 previously unpublished South Asian genomes.

### Sequencing, filtering, alignment, and variant calling

Illumina short reads were mapped to the reference genome (build GRCh38) using BWA-MEM^26^. We then used GATK4^27^ for base quality score recalibration, indel realignment, duplicate removal, variant quality score recalibration, variant discovery, and joint genotyping, using the GATK Best Practices recommendations^28,29^. We then removed variants that were monomorphic or were not annotated as PASS (i.e., not VQSRTrancheSNP99.90to100.00), and converted genotype calls with genotype quality (GQ) score <20 to missing data. Finally, we removed any variants with a missing genotype rate of >30%. Note that this more permissive missing genotype cutoff was used because of the presence of several hundred lower-coverage genomes in our initial call set. Most of these genomes were subsequently filtered out during the sample QC process described below.

### Comparison with 1000 Genomes Data

We downloaded genotype calls from the high-coverage 1000 Genomes Project data from http://ftp.1000genomes.ebi.ac.uk/vol1/ftp/data_collections/1000G_2504_high_coverage/working/20190425_NYGC_GATK/. We then used the same filters as described above, except with a genotype quality filter of ≥40. Then, for 22 individuals that were sequenced independently but were contained in both our call set and the 1000 Genomes Project (high-coverage) call set, we tabulated the non-reference discordance rate of the filtered genotype calls in the two datasets. Results are summarized in Supplementary Table 1.

### Sample QC and identification of first-degree relative pairs

We used KING^30^ to identify close relatives in our data. We labeled pairs of individuals as duplicates or first-degree relatives if the estimated kinship coefficients were >0.4 and [0.177, 0.4] respectively. Note that consanguinity should not affect the expected kinship coefficient for a given level of relatedness, though it likely affects the variance. We then removed samples in the following order:

1. All samples with duplicates from another population.
2. For remaining duplicate pairs in the same population, the individual with more missing data.
3. Individuals that have more than one first-degree relative.
4. Individuals with genotype calls at <90% of all SNPs.
5. For the remaining first-degree relative pairs, the individual with more missing data.

Removal of close relatives was performed so that our cohorts more closely represented randomly sampled individuals from the population for our analyses. Additional removal of second-degree relatives did not qualitatively change any of our results (J.D.W., unpublished data). After filtering, we retained 6442 genomes for downstream analyses.

### Phasing

The collection of 6442 individuals described above was computationally phased using eagle2^31^, with the default options (which includes allowing eagle2 to impute sporadic missing genotypes). We used the GRCh38-based genetic map provided with the eagle2 distribution for the phasing. We also used the same workflow to construct a reference panel consisting of only the South Asian medical cohorts, which was used in the design of the SARGAM array.

### Population structure (Fst, PCA, UMAP, and Admixture)

We used *plink* version 1.9^32^ to conduct Principal Components analysis. We filtered SNPs to have a MAF > 0.01, and LD-pruned using an *r*^2^ threshold of 0.2. We then created PCA plots after removing the pruned SNPs with the *variant-weights* modifier in *plink1.9*. These analyses were performed separately for different groups of individuals after the removal of first-degree relative pairs and low-coverage samples as described above.

UMAP projection was performed using the protocol and script published by Diaz-Papkovich et al.^33^. Fifteen principal components were used to generate the two-dimensional UMAP projection. Based on visualization and separation of known population groups in the Birbhum Cohort, we chose the key parameter settings as follows: number of neighbors (NN) of 15 and minimum distance (MD) of 0.5.

The ADMIXTURE^15^ analysis was performed using Version 1.3.0 of the software (http://www.genetics.ucla.edu/software/admixture). We used SNPs with MAF > 0.01, with a call rate >99.9%, and LD-pruned using a 50-SNP sliding window and variance inflation factor threshold of 2. The number of components K was optimized to minimize the cross-validation error using Chromosome 21. The optimal K for the Birbhum Cohort was 4, but for the larger South Asian and global samples, the cross-validation error continued to decrease even for a large *K* (*K* = 40). Thus, we chose to present the results at *K* = 12 which is the same number of components used in the GenomeAsia 100 K Pilot study^13^.

Weir and Cockerham weighted F_ST_ estimates were calculated using VCFtools^34^ Version 0.1.17. Only MAF-filtered and LD-pruned markers, as described above, were used. Samples that were related or did not pass QC were excluded.

### Rare homozygotes

For each population considered, we stratified variants according to the MAF in the specific population and tabulated the number of rare homozygotes and the expected number of rare homozygotes for each MAF category, assuming random mating. We then further stratified these results (Fig. 2c, d) by classifying some individuals as “inbred” (i.e., offspring of third-degree relatives or closer) or “outbred” (i.e., offspring of sixth-degree relatives or more distant), using the estimation process described below.

### Runs of homozygosity (ROH)

We used PLINK version 1.9^32^ to identify ROH in our data. We used the default parameter settings, except for the following:

--homozyg --homozyg-kb 500 --homozyg-window-snp 100 --homozyg-window-het 2 --homozyg-window-missing 20 --maf 0.001

We then converted the lengths of all ROHs into genetic distances, using a genetic map first created by Adam Auton and downloaded from the Beagle website at https://bochet.gcc.biostat.washington.edu/beagle/genetic_maps/

We required ROHs to have a minimum physical length of 500 Kb and a minimum genetic distance of 1 cM to be included in our analyses.

### Estimating the degree of inbreeding

We used a summary likelihood approach for estimating the degree of inbreeding from an individual’s ROH tracts. We focused on the longest ROHs to provide better power for distinguishing ROHs that arise due to endogamy versus ones that arise due to very recent inbreeding. Specifically, we tabulated (1) the number of ROHs longer than 10 cM, and (2) the sum of the genetic lengths of the 10 longest ROHs for each individual. Simulations suggest that these two summaries are slightly more informative than other, similar summaries based on the number or length of the longest ROH tracts (Supplementary Fig. 11). Concurrently, we simulated the distribution of ROH lengths expected under various degrees of inbreeding (see section directly below), ranging from the offspring of second-degree relatives (e.g., uncle–niece) to the offspring of sixth-degree relatives (e.g., half second cousins). Then, for each individual, we estimated the probability of observing the two ROH summaries (within 1% for the 10 longest ROHs) as a function of the degree of inbreeding. We then assigned the degree of inbreeding with the highest likelihood for each individual, treating sixth-degree relatives to be “unrelated”.

### Simulating expected ROH size distribution under inbreeding

We utilize the model of Clark^35^ for simulating the distribution of autozygous segments expected under a specific degree of inbreeding. Specifically, we assume that the genetic lengths of chromosomal segments inherited from particular paternal and maternal ancestors follow an exponential distribution with a mean equal to 100 cM divided by the total number of generations in the path from the proband back to the particular ancestors. For example, for an individual whose parents are first cousins, there are four paternal great-grandparents (and thus eight total paternal autosomal chromosomes three generations ago) and eight total maternal autosomal chromosomes that could be inherited at any particular genomic location. Of the 8 × 8 = 64 possible inheritance patterns, four result in consanguinity. We model an autosome’s ancestry as a series of blocks of ancestry, each with genetic length exponentially distributed with mean 100/6 cM, and with each block having a 4/64 = 6.25% chance of being autozygous. We use the genetic lengths of chromosomes estimated from the original deCODE genetic map^36^, and tabulate the number and size distribution of autozygous segments over 2 million simulations for each degree of consanguinity considered.

### Loss of function variants

A list of high-confidence LoF variants, including frameshift, splice-site, non-sense, start-loss, and stop-loss mutations, was obtained using the following criteria:

1. The LoF variants should be predicted as high confidence from the LOFTEE program^37^.
2. The LoF variants must fall within the high-confidence regions defined by the Genome-In-a-Bottle^38^ consortium (version – v.3.3.2).
3. The LoF variants cannot fall in segmental duplication regions of the genome (genomicSuperDups) as defined by the UCSC Genome Browser.
4. The Ensembl/GENCODE transcript with the highest expression among all the transcripts of the gene was retained. The expression value was obtained from the GTEx (Genotype-Tissue Expression) project (version 8) and averaged over all median tissue expressions.

### Burden test and association test

We analyzed a total of 2994 South Indian samples for which we had exome or whole-genome sequence data as well as blood lipid level measurements. Samples with extreme blood lipid values, defined as values outside of Q1–1.5 IQR/sqrt(n) and Q3 + 1.5 IQR/sqrt(n), were removed. Variant annotation was carried out using the Variant Effect Predictor^39^ annotated against Ensembl v75^40^. We considered only the LoF variants using the filters described above. Further, we removed samples and variants with poor call rates (>5% no calls) and only kept the variants with MAF < 0.1. For each sample, we combined the LoF variant dosages into a single burden within each gene and restricted the analysis to genes with at least 3 LoF variant carriers. Association analyses of quantitative traits were performed using linear regression on the burden score with age and sex as covariates.

### SARGAM array design

We partnered with Thermo Fisher Scientific to develop a custom genotyping array using their Axiom platform. The SARGAM (South Asian Research Genotyping Array for Medicine) assays a total of 639,029 SNPs, including 515,921 variants chosen to optimize imputation accuracy as well as 102,752 putatively functional variants that had a minor allele frequency of >0.1% in our medical cohorts. The imputation-based SNPs were chosen using an algorithm similar to the one described by Hoffmann et al.^41^, based on the South Asian medical cohort phased reference panel described above. The initial list of putative functional variants was obtained from a variety of sources, including this project, gnomAD^19^, the UK Biobank^42^ and properly consented MedGenome internal data.

We initially started with a larger list of 924,667 SNPs that were assayed on two custom test arrays that were then used to genotype 960 individuals from the GAsP2 study for quality control purposes. We removed SNPs that could not be genotyped accurately as well as low-priority variants to arrive at the final SARGAM array design.

### Imputation accuracy

We measure the performance of imputation in two complementary manners: imputation *r*^2^ and non-reference site concordance with the ground truth genotypes coming from 30× WGS data in 99 independently collected samples from across India (see ref. ^43^ for data description). Array genotypes were modeled by sub-sampling the 30X WGS data to the SARGAM and GSAv3 array markers. Imputation was performed using Beagle5.0^44^ with 1000 Genome Project Phase 3 and this study as reference panels. The imputation *r*^2^ is an output from Beagle^44^. Non-reference site concordance is the percentage of the variants called that match the ground truth at the zygosity level.

### Polygenic risk scores

#### South Asian samples

We used the genotype data of 1800 CAD cases and 1163 controls assayed using the Illumina GSA3 array covering more than 600,000 genome-wide markers^23^. All the samples had more than 95% of the markers successfully genotyped. We used Beagle5.0^44^ to impute all variants with minor allele count >4, using either the 1000 Genomes Project Phase 3 data or this study’s reference panel. The total numbers of imputed variants were 24,154,211 and 24,969,892, respectively. We used the markers reported by the CardiogramplusC4D consortium^45^, and the methods described in ref. ^23^. to construct PRSs.

#### UK Biobank European samples

We selected 2910 samples from the UK Biobank, comprising 1448 CAD cases and 1468 controls having European ancestry. The cases were selected with ICD-9 codes of 410.X, 411.0, 412.X, 429.79 or ICD-10 codes of I21.X, I22.X, I23.X, I24.1, I25.2. We used the imputed genetic data for the generation of PRSs. This research was conducted using the UK Biobank Resource under Application Number 42406.

### Reporting summary

Further information on research design is available in the Nature Portfolio Reporting Summary linked to this article.

## Supplementary information

Supplementary Information

Description of Additional Supplementary Files

Supplementary Data 1

Reporting Summary

## Data Availability

Information on variants and population-specific allele frequencies is available from https://browser.genomeasia100k.org. Raw fastq files for all Coriell samples are freely available from the SRA under NCBI BioProject PRJNA476341. Request forms for access to individual-level vcf files (for all newly generated genomes except those from PKN) are also available from https://browser.genomeasia100k.org. Researchers need to provide a brief description of what the data will be used for and to agree to standard terms of use as required by the consent forms. Individual-level data from the PKN samples are unavailable. Specifically, the IRB approval from the Center for non-Communicable Diseases in Pakistan does not permit the sharing of individual-level genetic data from the PKN samples due to concerns about privacy and potential identifiability of study participants and their close relatives. The SARGAM array is commercially available through MedGenome, Inc. Pricing inquiries can be made to sargam-array@medgenome.com

## Code availability

The code and workflow used for estimating the degree of consanguinity from the patterns of ROH are available at https://github.com/jd-wall/CALCROH.
