# Supplementary material for: South Asian medical cohorts reveal strong founder effects and high rates of homozygosity
Source: Nat Commun. 2023 Jun 8;14:3377. doi: 10.1038/s41467-023-38766-1 (PMC10250394; doi:10.1038/s41467-023-38766-1)

Supplementary Note 1 - Sample recruitment and informed consent procedures

Information on sample recruitment and informed consent for newly sequenced samples is described below separately by population.

BRB: The Society for Health and Demographic Surveillance (SHDS) enrolled participants from the Birbhum Health and Demographic Surveillance System study[1] for this project. The ethical approval for this study was obtained from the Ethics Committee of the SHDS. Participants were informed about the process and significance of the study and an informed and written consent was obtained prior to enrollment. The authors also declare that all procedures contributing to this work comply with the ethical standards of the relevant national and institutional committees on human experimentation and with the Helsinki Declaration of 1975, as revised in 2008.

BAN: Subjects were ascertained and samples collected as part of the Bangladesh Risk of Acute Vascular Events (BRAVE) study using procedures as described in reference 2. The BRAVE study was approved by the Ethical Review Committee at the International Centre for Diarrhoeal Disease Research, Bangladesh (icddr,b) under application number #PR-10006. Written informed consent was obtained from each participant prior to recruitment, including for use of stored samples for biochemical, genetic and other analyses. Data collected in this research are subject to the core data protection principles and requirements of the UK Data Protection Act 1998. The investigators and institutional review boards are committed to ensure that research is conducted according to the latest version of the Declaration of Helsinki, the Universal Declaration on the Human Genome and Human Rights adopted by UNESCO, and other relevant legislation.

BLR: Samples from type 2 diabetes patients undergoing treatment for diabetic retinopathy were obtained from the outpatient population of an eye hospital in Bangalore. The study was approved by the Institutional Ethics Committee of Narayana Nethralaya (# C/2014/07/04) and adhered to the tenets of the Declaration of Helsinki. All samples were obtained with written informed consent of the subjects.

COI: Clinically suspected DMD/BMD male patients from the state of Tamil Nadu (TN), India were received at MDCRC, (a non- profit organization dedicated to diagnosis, care and counseling of patients with Muscular Dystrophies: www.mdcrcindia.org) between 2006 to 2013, for molecular diagnosis. Patients were referred to MDCRC through an MDCRC Community Genetics initiative which relied on a community outreach program to identify, diagnose and counsel patients in rural districts of TN state. Some patients were also referred by hospitals and clinics in the state. An informed written consent was obtained from each patient or parent in the local language (Tamil), where appropriate, prior to inclusion in the study. The Institutional Review Board of Molecular

Diagnostics, Counseling, Care & Research Centre (MDCRC) reviewed and approved this study

MAA: Type 2 diabetic subjects were recruited from Dr. Mohan's Diabetes Specialties Centre, a large diabetes center in Chennai (formerly Madras) city in southern India, which has a population of about 6 million people. All patients underwent a structured assessment including detailed family history.

The samples were obtained under appropriate informed consent with study review and approval obtained from the Institutional Ethics Committee of the Madras Diabetes Research Foundation, Chennai, India. The reported investigations have been carried out in accordance with the principles of the Declaration of Helsinki.

Consented and de-identified patients' blood samples were used for extraction of DNA. EDTA anti-coagulated venous blood samples were collected from all study subjects, and the genomic DNA was isolated from whole blood by proteinase K digestion followed by phenol-chloroform extraction. Subsequently genomic DNA was precipitated in ethanol. The quality and quantity were assessed spectrophotometrically.

PKN: Subjects ascertained and samples collected by the Center for Non-Communicable Disease (CNCD) in Karachi Pakistan as described in reference 3. Ethical approval for the study was obtained from the IRB committee of the CNCD which is registered with the NIH (IORG005843, IRB00007048, FWA00014490). Permission was granted for the release of aggregated genetic data (e.g., allele frequencies), but not for the release of individual-level data.

BAL and PAR: DNA samples were collected by Syed Qasim Mehdi (deceased) with IRB approval from the University of Karachi, Pakistan.

BEB, CHB, CHS, DAI, GIH, ITU, JPN, MAS, PGL and STU: DNA samples from de-identified individuals from the International HapMap and 1000 Genomes Projects were purchased from the Coriell Institute for Medical Research. All raw reads generated from these samples (and previous ones in the first GenomeAsia publication) have been deposited in the Sequence Read Archive under accession number PRJNA476341 at https://www.ncbi.nlm.nih.gov/bioproject/476341.

AHI, BHA, BLM, MEO, MHB, MRT, PNB, PTL, RMG, SCH, SIK and SNT: DNA samples were collected by the National Institute of Biomedical Genomics (NIBMG) in West Bengal, India by Partha Majumder. Participant recruitment and collection of blood samples were approved by the Ethics Committees of Guru Nanak Dev University (Chandigarh), Research Society B. J. Wadia Hospital (Mumbai), Bharathiar University (Coimbatore), North-Eastern Hill University (Shillong) and the Indian Statistical Institute (Kolkata). A broad consent was taken from each participant for research use of the collected blood samples with appropriate anonymization.

Please note that the 99 whole genome sequences used for estimating imputation accuracy are available through the GenomeAsia portal but are described in reference 4.

Supplementary References

1. Ghosh, S., Barik, A., Majumder, S., Gorain, A., Mukherjee, S. Mazumdar, S., Chatterjee, K., Baumik, S. K., Bandyopadhyay, S. K., Satpathi, B., et al. (2015) Health and demographic surveillance system profile: the Birbhum population project (Birbhum HDSS). Int J Epidemiol, *44*, 98-107.

2. Chowdhury, R., Alam, D. S., Fakir, I. I., Adnan, S. D., Naheed, A., Tasmin, I., Monower, M. M., Hossain, F., Hossain, F. M., Rahman, M. M., et al. (2015) The Bangladesh Risk of Acute Vascular Events (BRAVE) study: objectives and design. Eur J Epidemiol, *30*, 577-687.

3. Saleheen, D., Zaidi, M., Rasheed, A., Ahmad, U., Hakeem, A., Murtaza, M., Kayani, W., Faruqui, A., Kundi, A., Zaman, K. S., et al. (2009) The Pakistan Risk of Myocardial Infarction Study: a resurce for the study of genetic, lifestyle and other determinants of myocardial infarction in South Asia. Eur J Epidemiol, *24*, 329-338.

4. Kukkle, P. L., Thenral, S. G., Parkinson Research Alliance of India (PRAI), Chaudhary, R., Sathirapongsasuti, J. F., Goyal, V., Kandadai, R. M., Kumar, K., Borgohain, R., Mukherjee, A., et al. (2022). Genome-wide polygenic score predicts large number of high risk individuals in monogenic undiagnosed Young Onset Parkinson's Disease (YOPD) patients in India. Advanced Biology, *6*, e2101326.

Supplementary Table 1. Non-reference discordance rate for selected samples that overlap between our data set and the 1000 Genomes Project data.

Sample ID	Population	Non-reference sites	Discordant sites
HG03593	BEB	2559776	302
HG03594	BEB	2554669	271
NA18525	CHB	2477292	765
NA18528	CHB	2487659	651
HG00759	DAI[1]	2487841	458
HG00766	DAI[1]	2482621	515
HG00097	GBR	2488235	351
HG00099	GBR	2480926	420
NA20847	GIH	2546961	241
NA20849	GIH	2545555	342
HG03714	ITU	2560067	287
HG03716	ITU	2550291	516
NA18939	JAP[2]	2488880	180
NA18941	JAP[2]	2481018	271
HG02072	KIN[3]	2499035	326
HG02073	KIN[3]	2487351	274
HG03631	PJL	2552091	200
HG03634	PJL	2547690	290
HG03642	STU	2558974	439
HG03672	STU	2527124	405
NA18510	YOR	3027593	765
NA18908	YOR	3031390	817

[1] Population code is CDX in the 1000 Genomes Project; [2] Population code is JPT in the 1000 Genomes Project; [3] Population code is KHV in the 1000 Genomes Project

Supplementary Table 2: Number of well-imputed variants with different reference panels

Imputation Reference	# Imputed sites	Common sites with Cardiogram study (~6.6million)
GAsP2	24,969,890	5,112,560
1000G	24,154,211	3,832,179

Supplementary Figure 1 - The UMAP plot of all 6,442 GenomeAsia samples, including the 4,807 South Asian samples. Colors depict the sample origin/location. The full names for the three-letter codes can be found in Supplementary Table 1.

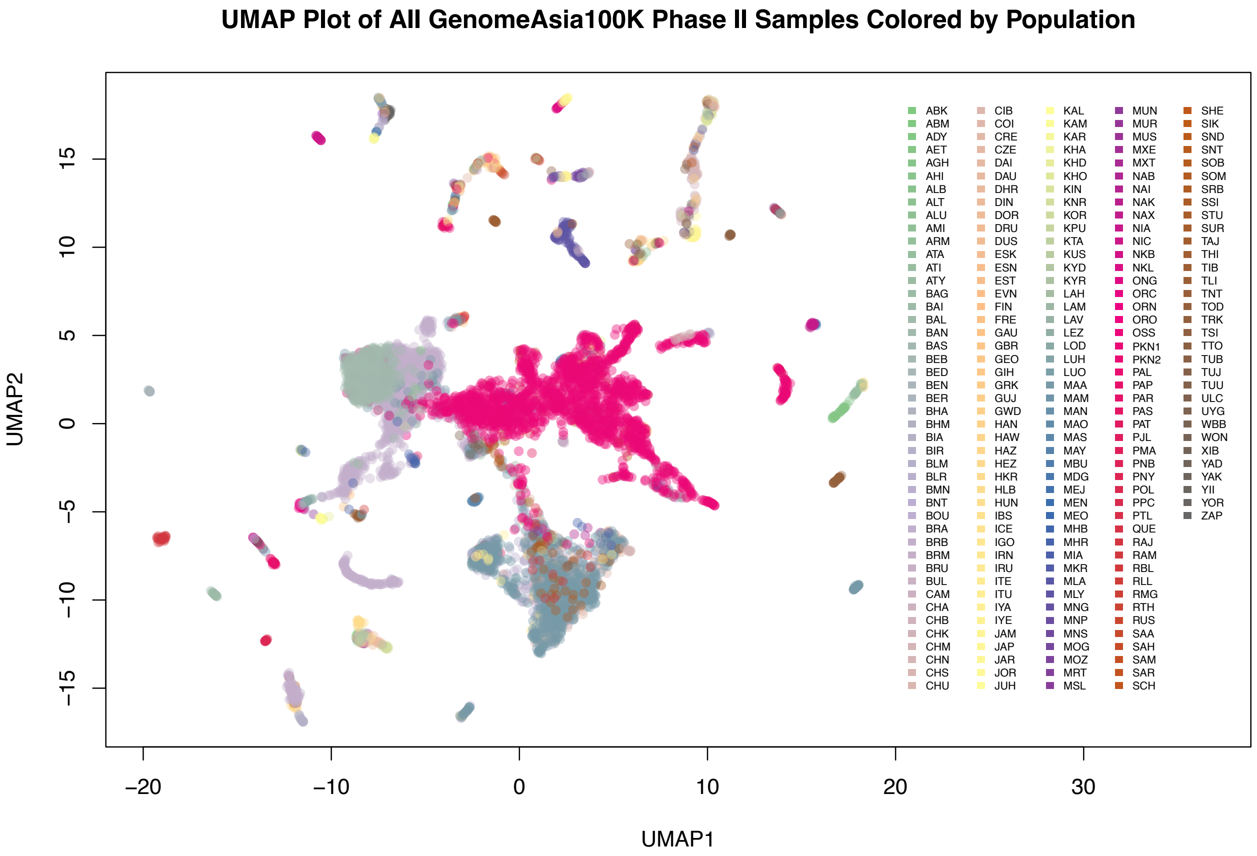

Supplementary Figure 2 - Additional ADMIXTURE plots for (A-B) Birbhum cohort and (C-D) all major South Asian cohorts. Cross-validation (CV) error was used to determine the optimal number of ADMIXTURE components (K). For the Birbhum cohort, K=4 appears optimal and resulted in the ADMIXTURE plot (B), which is organized by the average group proportion of the "red" component. (C) The CV error did not reach an optimal point even after increasing the K to 20. For interpretability, we depicted ADMIXTURE plot at K=12 (D)

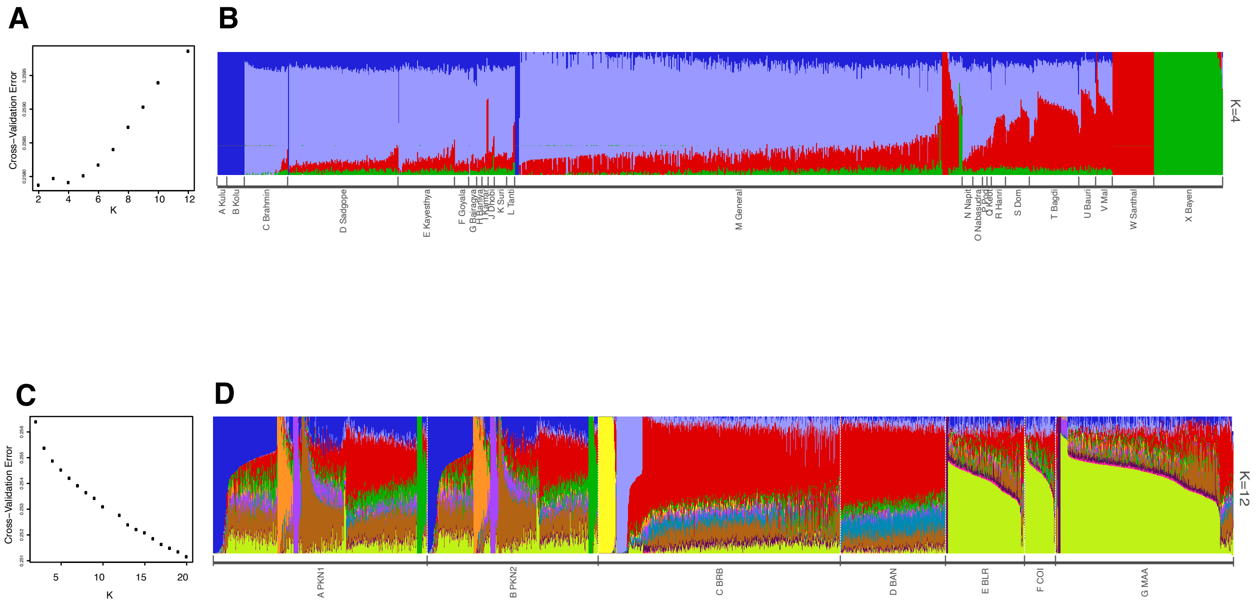

Supplementary Figure 3 - PCA plots of all 6,442 GenomeAsia samples. (A) First two eigenvectors plotted for the analysis of all GenomeAsia samples. (B) First two eigenvectors plotted for the analysis of all GenomeAsia samples of South Asian individuals including both isolated population groups and patient populations. (C) First two eigenvectors for GenomeAsia South Asian patient population samples. Colors depict the sample origin/location according to the legend in each panel. The key for the three-letter codes can be found in Supplementary Note 1.

A

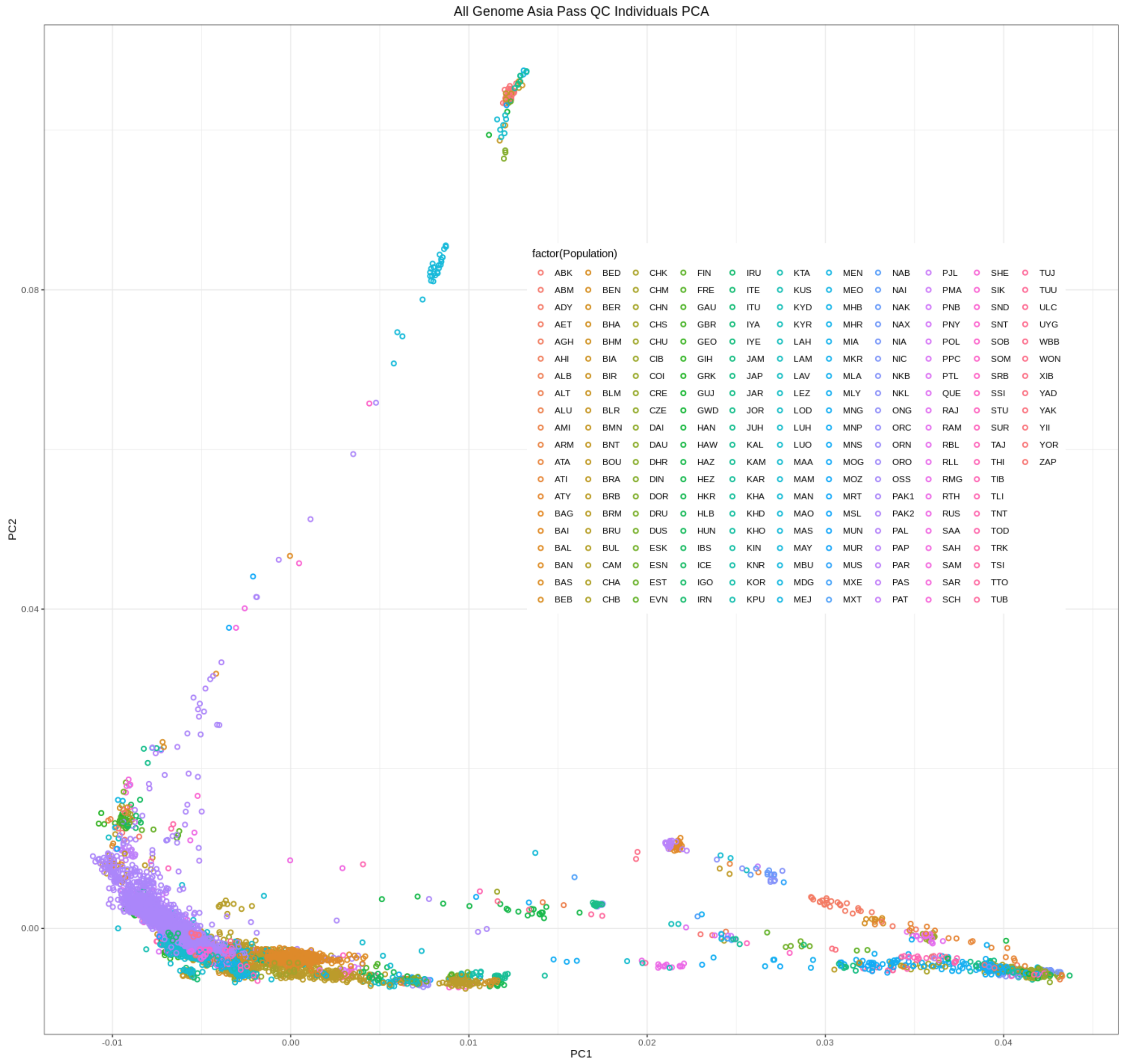

B

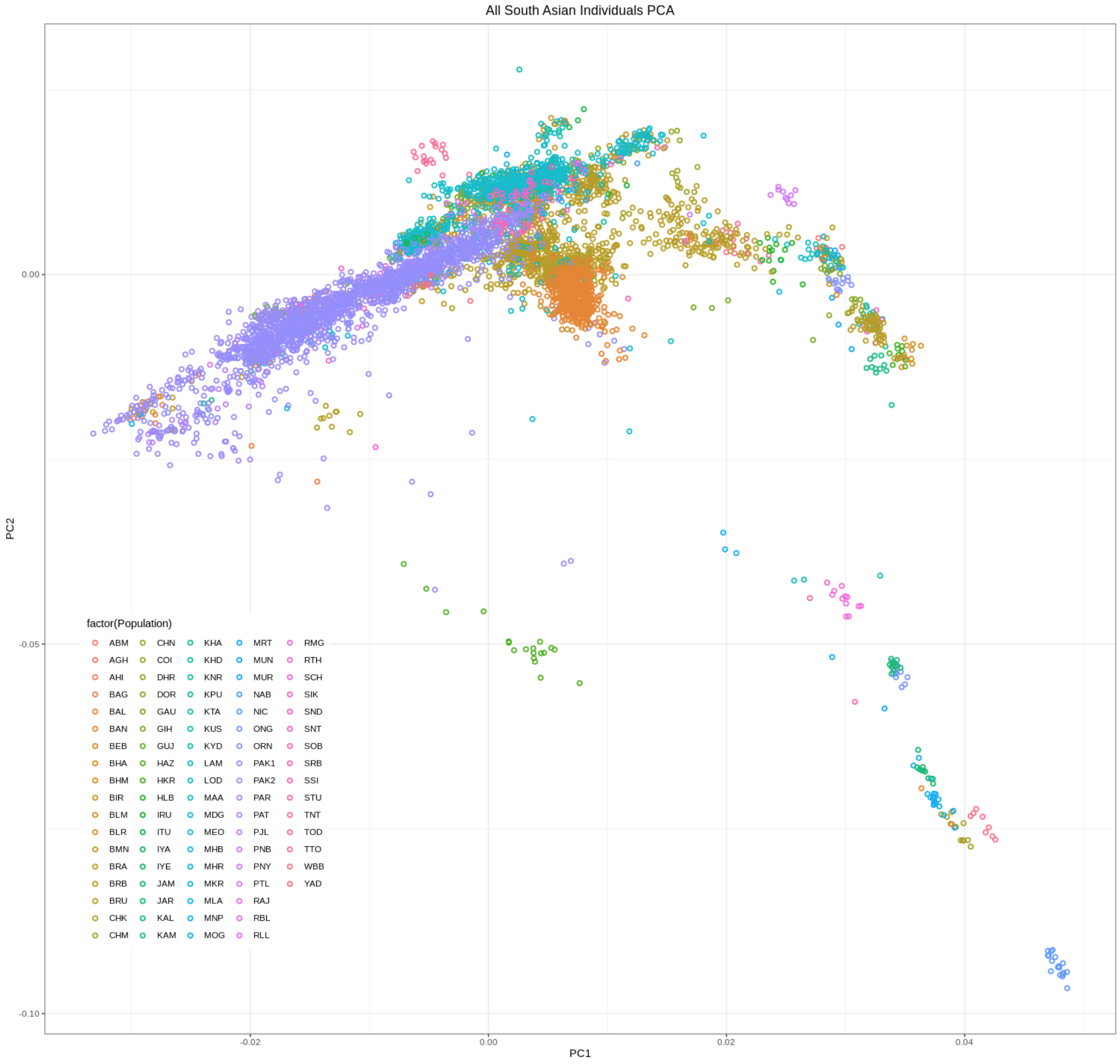
All South Asian Individuals PCA
PC2
PC1
0.00
-0.05
-0.10
-0.02
0.00
0.02
0.04
factor(Population)
ABM AGH AHI BAG BAL BAN BEB BHA BHM BIR BLM BLR BMN BRA BRB BRU CHK CHM
CHN COI DHR DOR GAU GIH GUJ HAZ HKR HLB IRU ITU IYA IYE JAM JAR KAL KAM
KHA KHD KNR KPU KTA KUS KYD LAM LOD MAA MDG MEO MHB MHR MKR MLA MNP MOG
MRT MUN MUR NAB NIC ONG ORN PAK1 PAK2 PAR PAT PJL PNB PNY PTL RAJ RBL RLL
RMG RTH SCH SIK SND SNT SOB SRB SSI STU TNT TOD TTO WBB YAD

C

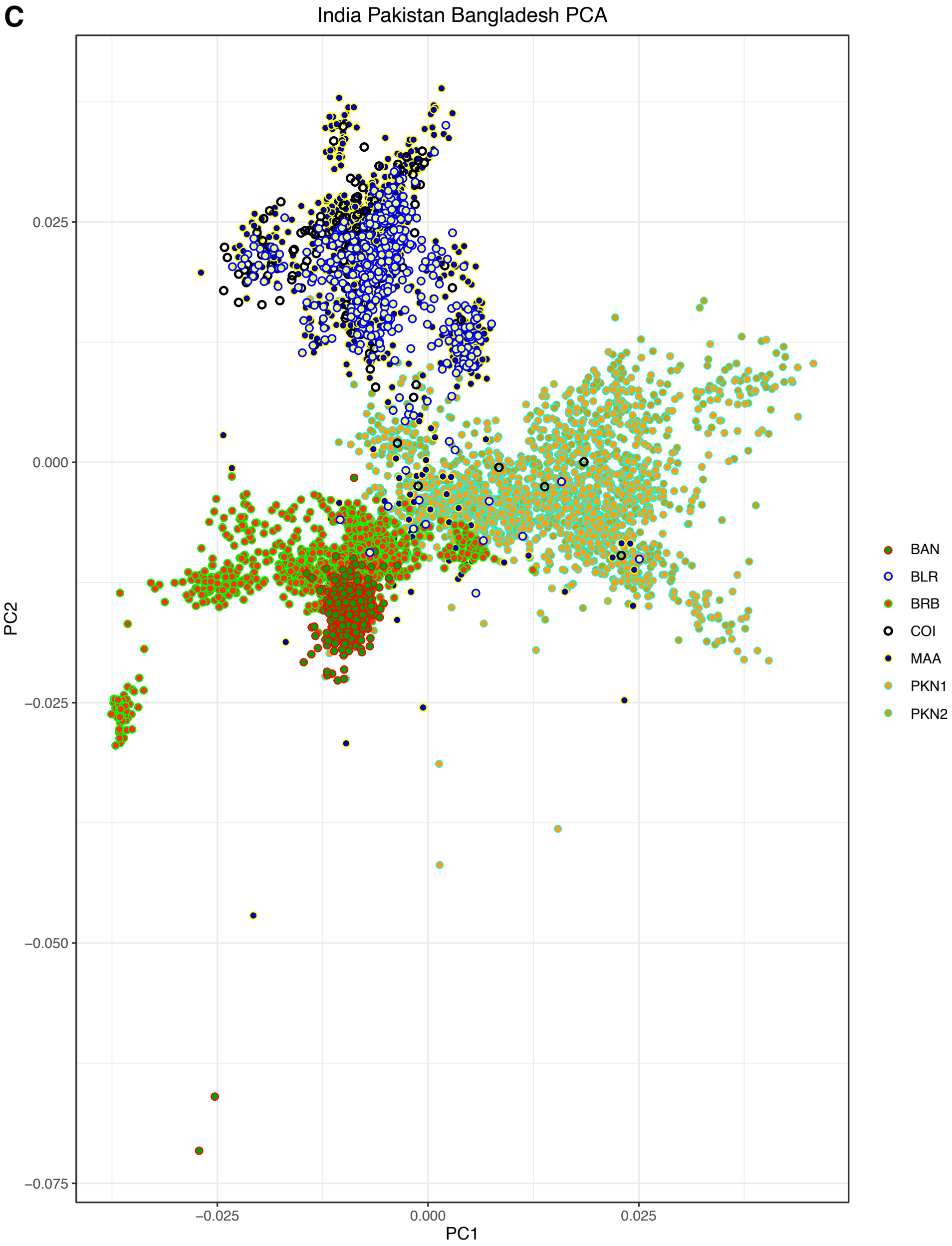
India Pakistan Bangladesh PCA
PC2
PC1
0.025
0.000
−0.025
−0.050
−0.075
−0.025
0.000
0.025
BAN
BLR
BRB
COI
MAA
PKN1
PKN2

Supplementary Figure 4 - Fst plot showing the divergence among the major South Asian groups and British in England and Scotland (GBR), as a representation of non-Finnish Europeans. This confirms a closer relationship between Pakistanis and Europeans and among South Indians (MAA, BLR, and COI).

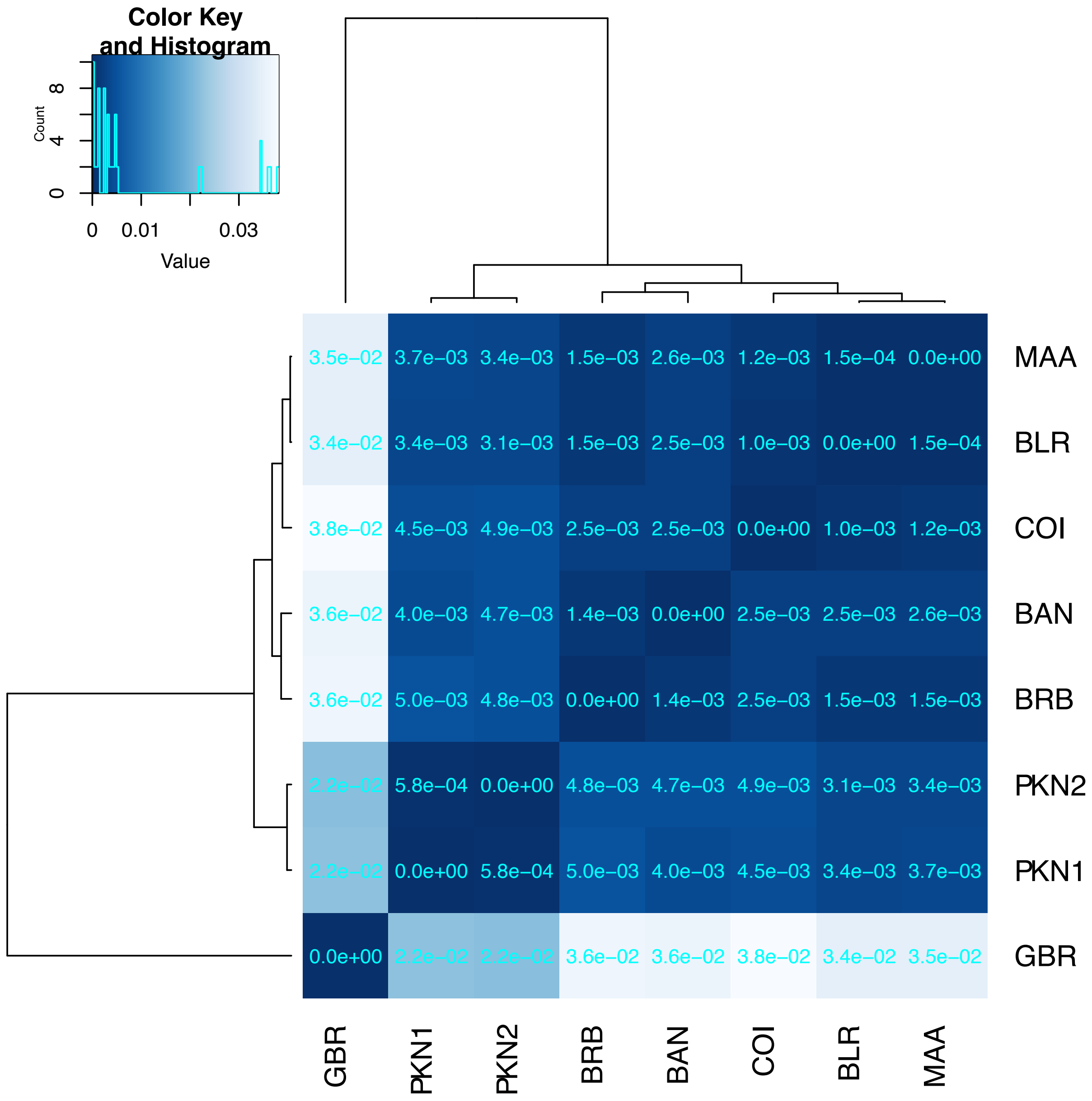

Supplementary Figure 5 - Genetic estimates of consanguinity for general caste Muslims from Birbhum, stratified by self-reported consanguinity. 2nd = parents are 2nd degree relatives, 3rd = parents are 3rd degree relatives, 4th = parents are 4th degree relatives, 5th = parents are 5th degree relatives, Unr = parents are 6th degree relatives or more distantly related to each other.

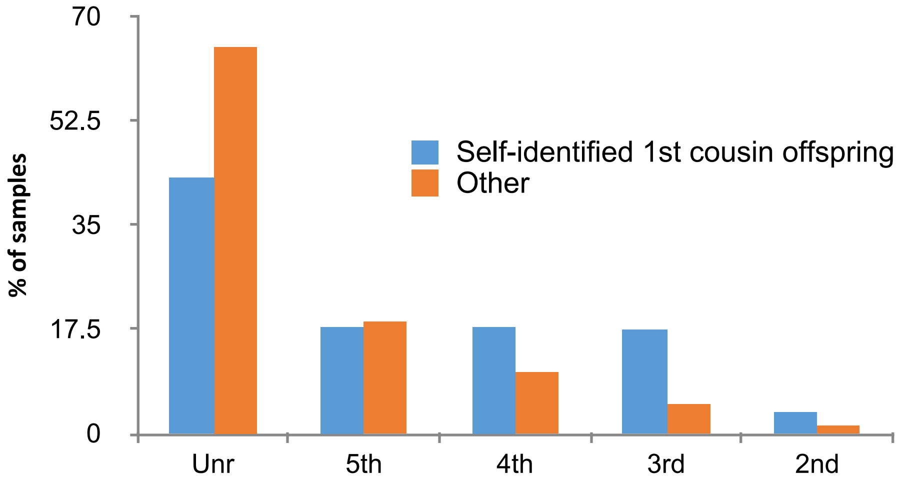

Supplementary Figure 6 - Ridgeplots (analogous to Figure 2f) showing the stratification of Figure 2e's SOI (South Indian) and BAN (Bangla) plots into groups with different estimated degrees of inbreeding.

A) BAN (Bangla)

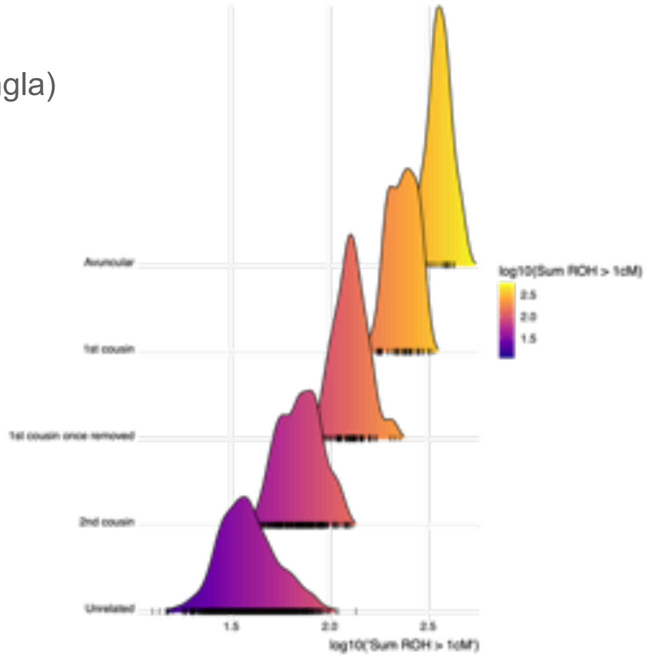

B) SOI (South Indian)

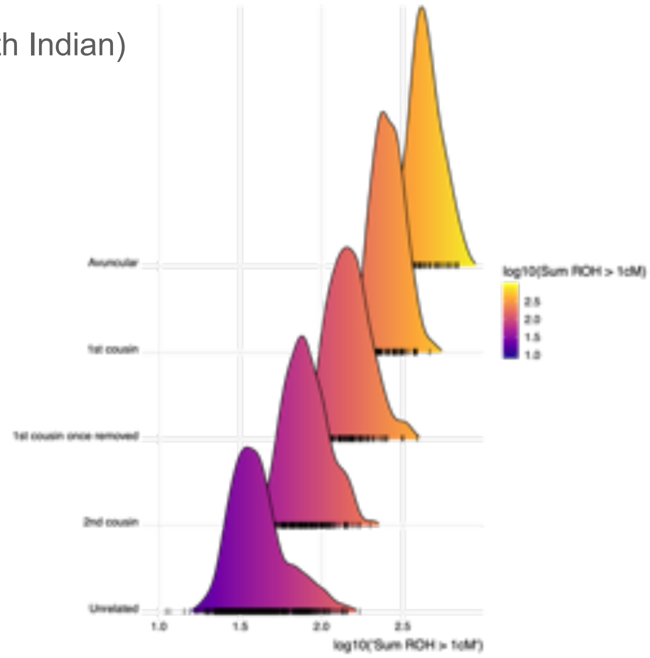

Supplementary Figure 7. Homozygous loss of function gene space by population. Each square represents a distinct gene and is colored by its maximum LoF burden using the same color scheme as **Figure 3b**. Genes are separated by groups in which they are found, similar to **Figure 3b** (as marked below). For each group, the relative order of the genes is the same as in **Figure 3b**. This is why each group has a patchwork of colored and blank squares.

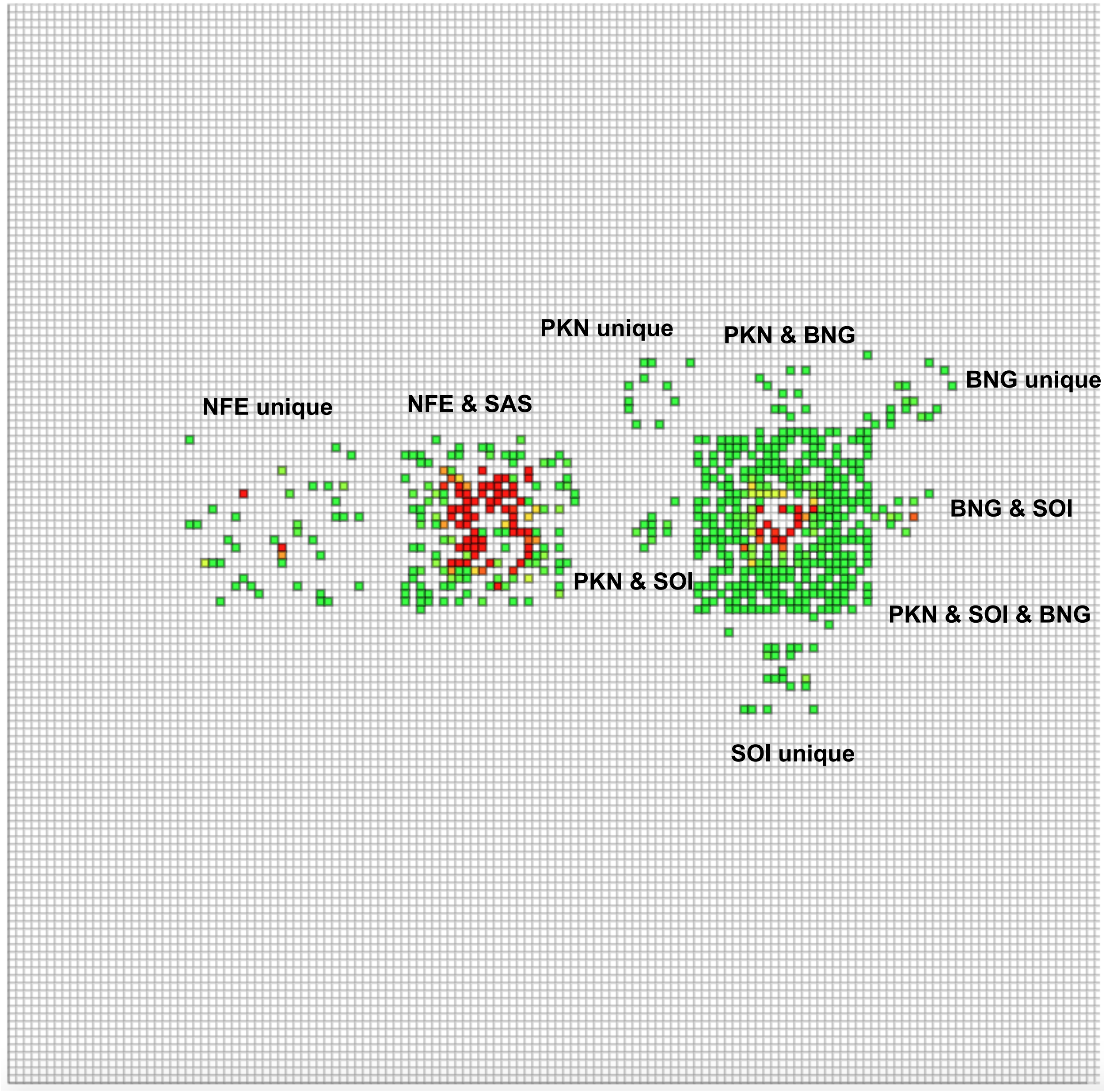

Supplementary Figure 8. Proportion of rare, homozygous pLoF mutations that fall within runs of homozygosity of different sizes, stratified by estimated degree of consanguinity. Mutations that do not fall within an ROH are put in the “<1 cM” category. Unrelated = parents are 6th degree relatives or more distantly related to each other, 2nd cousins = parents are 5th degree relatives, 1st cousins once removed = parents are 4th degree relatives, 1st cousins = parents are 3rd degree relatives, Avuncular = parents are 2nd degree relatives.

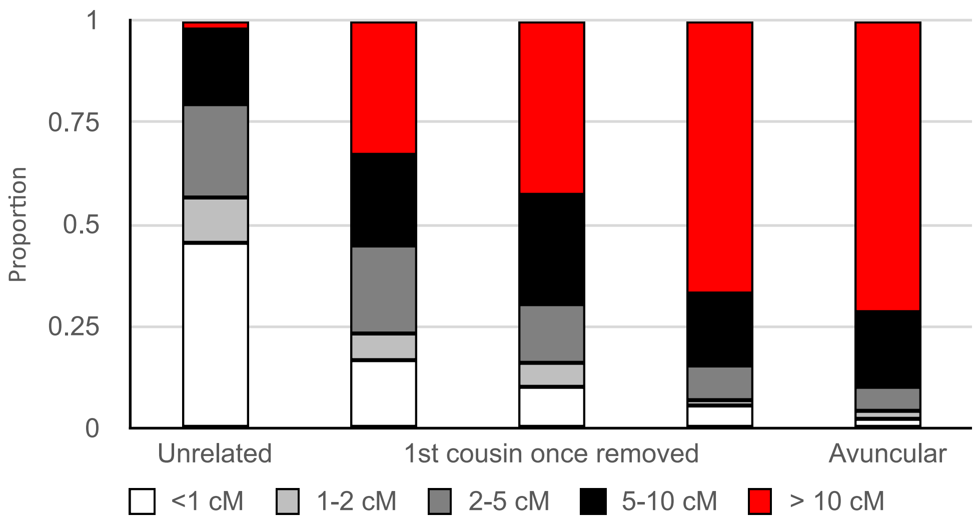

Supplementary Figure 9 - Population-specific HBB missense and LoF alleles can be seen on the UMAP plot. (A) A missense variant rs334 is known to cause sickle cell anemia and is prevalent in central Indian and west Bengalis. (B-E) LoF variants in HBB gene cause beta thalassemia and are found mostly in northern Indians and Pakistanis.

A

UMAP Plot of All GenomeAsia100K Phase II Samples Colored by Allele Count of rs334_A

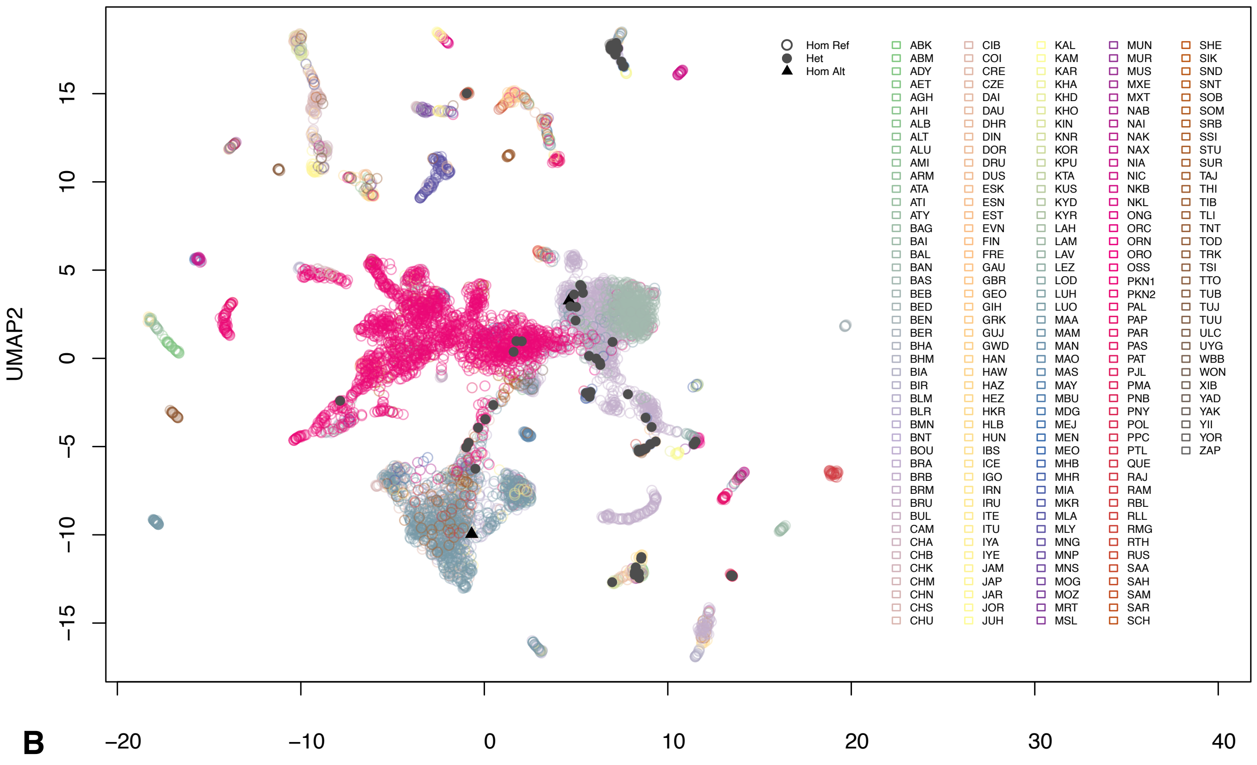

B

UMAP Plot of All GenomeAsia100K Phase II Samples Colored by Allele Count of HBB_LoF_burden

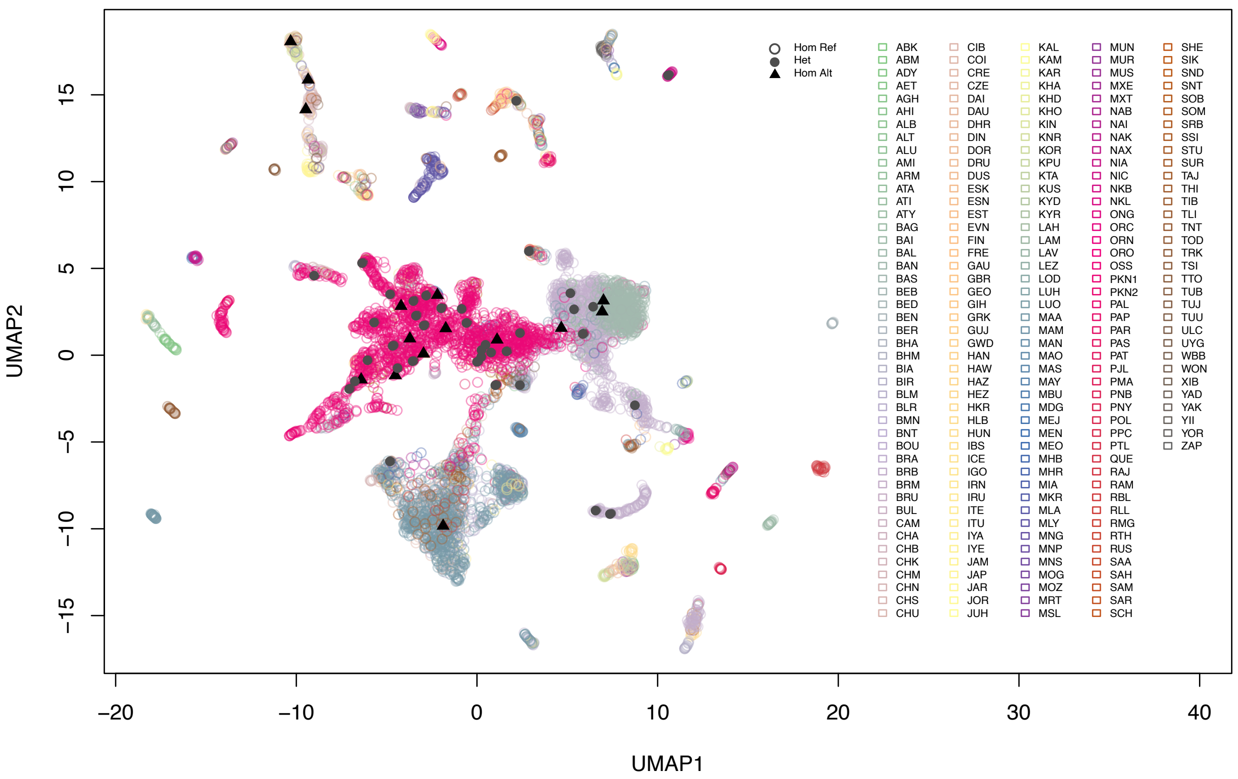

C

UMAP Plot of All GenomeAsia100K Phase II Samples Colored by Allele Count of chr11_5226765_AG_._.

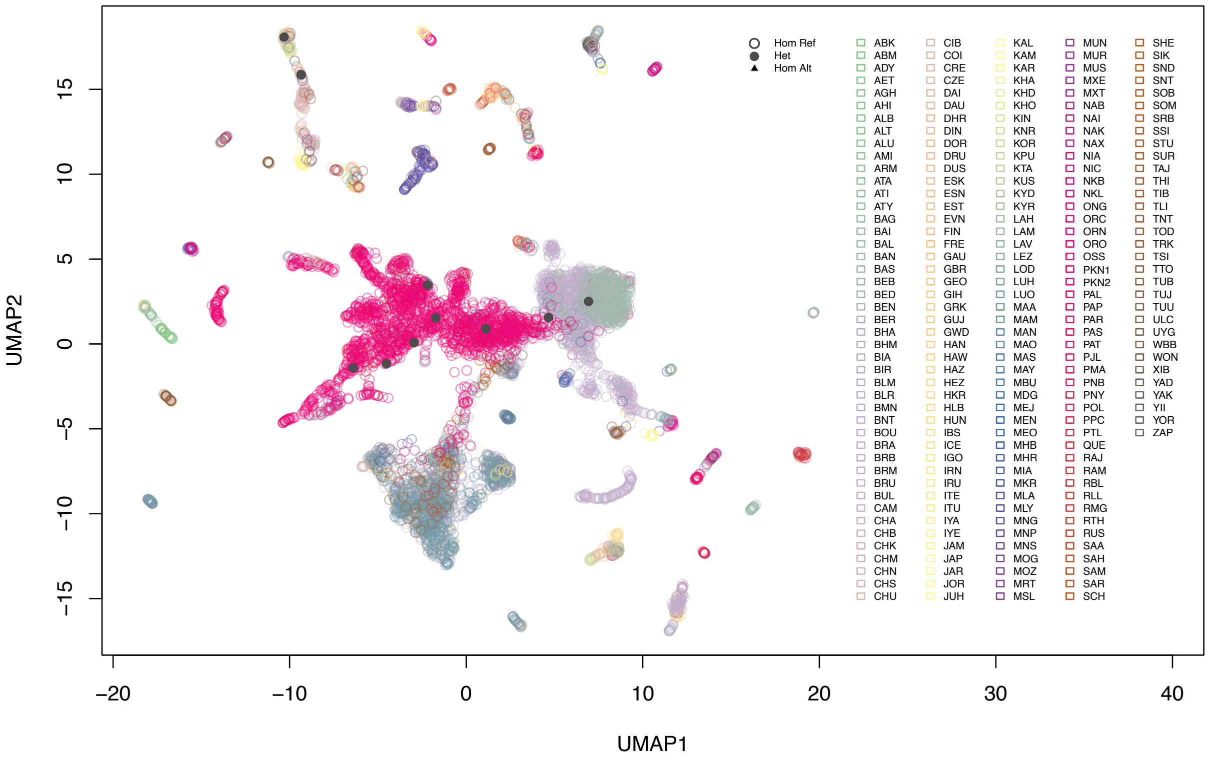

D

UMAP Plot of All GenomeAsia100K Phase II Samples Colored by Allele Count of chr11_5226765_AG_._.

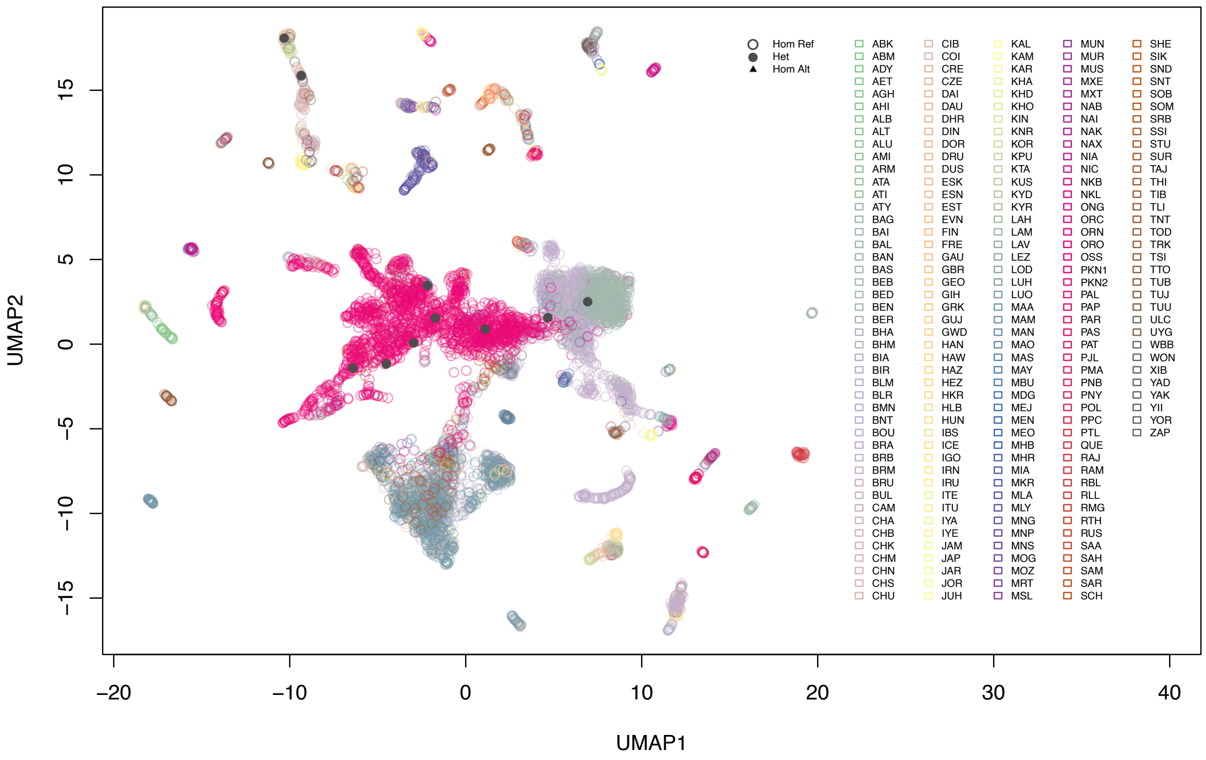

E

UMAP Plot of All GenomeAsia100K Phase II Samples Colored by Allele Count of chr11_5226975_C_T_T

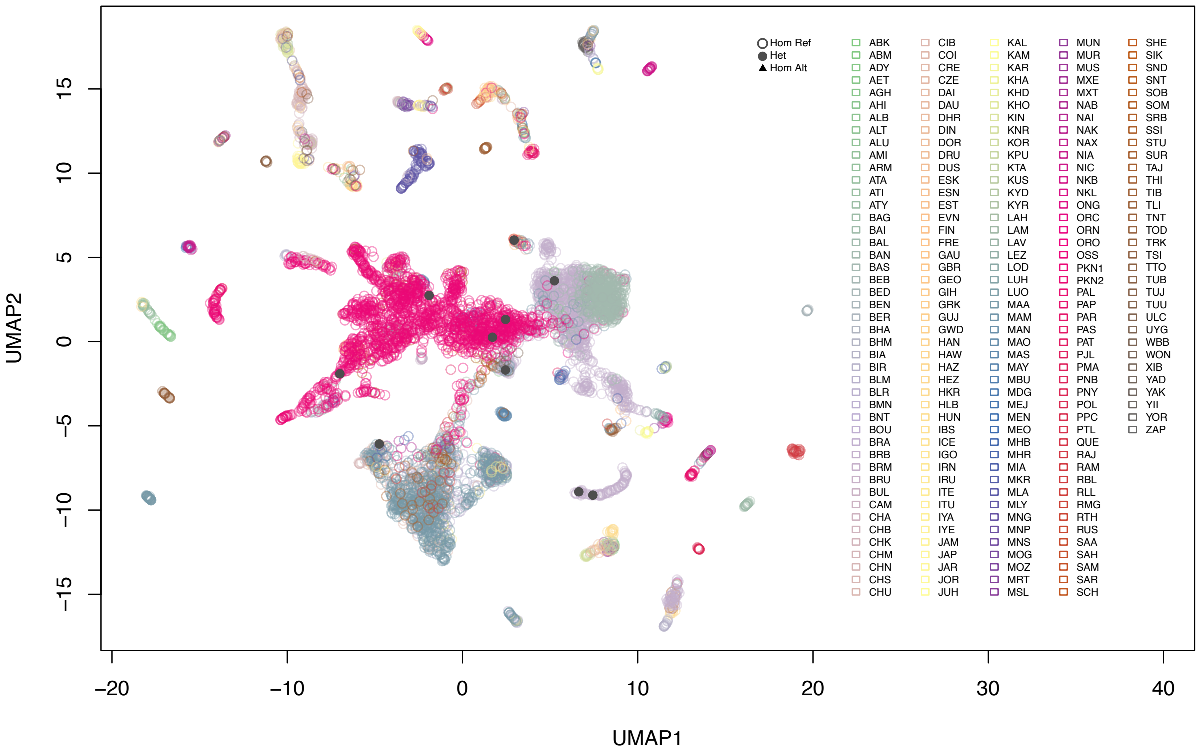

Supplementary Figure 10 - Increased number of CAD PRS variants that can be imputed by GAsP2 and 1000G panels. The GenomeAsia imputation panel allows for more variants to be imputed (25.0M) as compared to the 1000 Genome panel (24.2M). Among those imputable variants, significantly more overlap the CAD PRS model, contributing significantly to the improvement of the PRS.

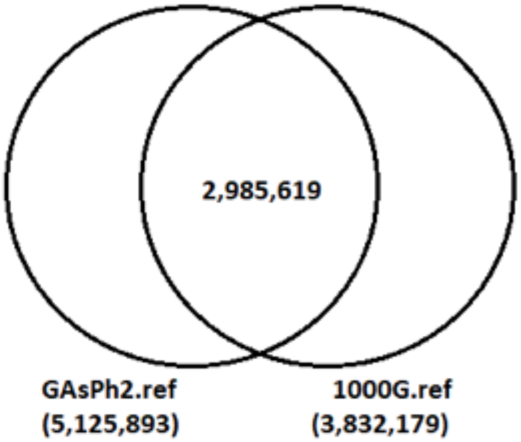

Supplementary Figure 11 – Comparison of different ROH-based summary statistics in estimating the degree of consanguinity. Simulations were run using the scheme described in the main text to estimate the relevant likelihoods (10^6 simulations for each parameter combination). All approaches used the number of ROH at least 10 cM in length as one summary. The other summary used was the sum of the genetic lengths of the top 10 ROH (black), the average genetic length of all ROH ≥ 5 cM (green), the sum of genetic lengths of all ROH ≥ 5 cM (grey), and the sum of genetic lengths of all ROH ≥ 10 cM (yellow). Accuracy was then assessed by simulating ROH lengths for the offspring of 1st cousins or 2nd cousins, and tabulating how likely each summary likelihood approach was to estimate the correct degree of consanguinity.

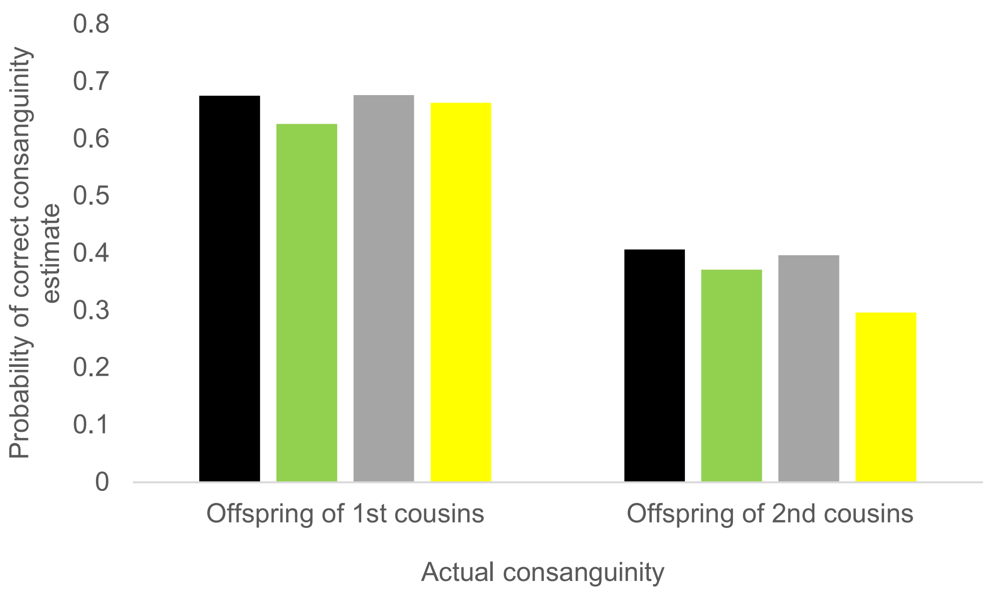

Supplement: Supplementary file 1 — Supplementary Information [file 41467_2023_38766_MOESM1_ESM.pdf]
