# Supplementary material for: South Asian medical cohorts reveal strong founder effects and high rates of homozygosity
Source: Nat Commun. 2023 Jun 8;14:3377. doi: 10.1038/s41467-023-38766-1 (PMC10250394; doi:10.1038/s41467-023-38766-1)
Supplement: Supplementary file 2 — Description of Additional Supplementary Files [file 41467_2023_38766_MOESM2_ESM.pdf]

Description of Additional Supplementary Files

File Name: Supplementary Data 1
Description: Basic metadata about samples and populations. a) List of sample IDs and population labels; b) List of population sample sizes and three letter codes.
