# Supplementary material for: South Asian medical cohorts reveal strong founder effects and high rates of homozygosity
Source: Nat Commun. 2023 Jun 8;14:3377. doi: 10.1038/s41467-023-38766-1 (PMC10250394; doi:10.1038/s41467-023-38766-1)
Supplement: Supplementary file 4 — Reporting Summary [file 41467_2023_38766_MOESM4_ESM.pdf]

nature portfolio

Corresponding author(s): Jeff Wall

Last updated by author(s): Apr 28, 2023

Reporting Summary

Nature Portfolio wishes to improve the reproducibility of the work that we publish. This form provides structure for consistency and transparency in reporting. For further information on Nature Portfolio policies, see our Editorial Policies and the Editorial Policy Checklist.

Statistics

For all statistical analyses, confirm that the following items are present in the figure legend, table legend, main text, or Methods section.

n/a	Confirmed	
☐	☒	The exact sample size (n) for each experimental group/condition, given as a discrete number and unit of measurement
☐	☒	A statement on whether measurements were taken from distinct samples or whether the same sample was measured repeatedly
☐	☒	The statistical test(s) used AND whether they are one- or two-sided<br>*Only common tests should be described solely by name; describe more complex techniques in the Methods section.*
☐	☒	A description of all covariates tested
☐	☒	A description of any assumptions or corrections, such as tests of normality and adjustment for multiple comparisons
☐	☒	A full description of the statistical parameters including central tendency (e.g. means) or other basic estimates (e.g. regression coefficient) AND variation (e.g. standard deviation) or associated estimates of uncertainty (e.g. confidence intervals)
☐	☒	For null hypothesis testing, the test statistic (e.g. F, t, r) with confidence intervals, effect sizes, degrees of freedom and P value noted<br>*Give P values as exact values whenever suitable.*
☒	☐	For Bayesian analysis, information on the choice of priors and Markov chain Monte Carlo settings
☒	☐	For hierarchical and complex designs, identification of the appropriate level for tests and full reporting of outcomes
☒	☐	Estimates of effect sizes (e.g. Cohen's d, Pearson's r), indicating how they were calculated

Our web collection on statistics for biologists contains articles on many of the points above.

Software and code

Policy information about availability of computer code

Data collection	No software was used.
Data analysis	GATK4 (Best practices workflow), KING (v2.2.4), Eagle (v2.4.1), plink (vl.9), UMAP script (from reference 21), ADMIXTURE (vl.3.0), VCFtools (v0.1.17), LOFTEE (vl.0.3), Beagle (vS.0), custom pipeline for estimating consanguinity, available on Github at https://github.com/jd-wall/CALCROH

For manuscripts utilizing custom algorithms or software that are central to the research but not yet described in published literature, software must be made available to editors and reviewers. We strongly encourage code deposition in a community repository (e.g. GitHub). See the Nature Portfolio guidelines for submitting code & software for further information.

Data

Policy information about availability of data

All manuscripts must include a data availability statement. This statement should provide the following information, where applicable:

- Accession codes, unique identifiers, or web links for publicly available datasets
- A description of any restrictions on data availability
- For clinical datasets or third party data, please ensure that the statement adheres to our policy

Information on variants and population-specific allele frequencies are available from https://browser.genomeasia100k.org. Raw fastq files for all Coriell samples are freely available from the SRA under NCBI BioProject PRJNA476341. Request forms for access to individual-level vcf files (for all newly generated genomes except

those from PKN) are also available from https://browser.genomeasia100k.org. Researchers need to provide a brief description of what the data will be used for and to agree to standard terms of use as required by the consent forms. The IRB approval from the Center for non-Communicable Diseases in Pakistan does not permit the sharing of individual level genetic data from the PKN samples due to concerns about privacy and potential identifiability of study participants and their close relatives.
The SARGAM array is commercially available through MedGenome, Inc. Pricing inquiries can be made to sargam-array@medgenome.com

Research involving human participants, their data, or biological material

Policy information about studies with human participants or human data. See also policy information about sex, gender (identity/presentation), and sexual orientation and race, ethnicity and racism.

Reporting on sex and gender	All recruitment and analyses were performed without regard to sex or gender. Neither sex nor gender were tabulated or used in any way.
Reporting on race, ethnicity, or other socially relevant groupings	Study participants were grouped based on sampling location for the primary analyses: Pakistanis (PKN), Bengalis (BNG, from both India and Bangladesh) and South Indians (SOI). Secondary analyses utilized self-described caste or group identify from the participants.
Population characteristics	Not applicable.
Recruitment	Participants were recruited as part of ongoing demographic studies (Birbhum, West Bengal, India) or as volunteers from the health care delivery system (Bangladesh, South India and Pakistan). We recruited all individuals willing to participate in the study. While there is no obvious bias (in terms of who participated and any characteristic studies), there is always a potential for bias associated with individuals more likely to seek health care and/or in agreeing to participate in the study.
Ethics oversight	Due to the hostile politics involving two of the countries included in our study (specifically, India and Pakistan), we did not have a single IRB covering all of the participants. The individual IRBs that approved specific cohorts for inclusion in our work are all listed in Supplementary Note 1.

Note that full information on the approval of the study protocol must also be provided in the manuscript.

Field-specific reporting

Please select the one below that is the best fit for your research. If you are not sure, read the appropriate sections before making your selection.

☒ Life sciences ☐ Behavioural & social sciences ☐ Ecological, evolutionary & environmental sciences

For a reference copy of the document with all sections, see nature.com/documents/nr-reporting-summary-flat.pdf

Life sciences study design

All studies must disclose on these points even when the disclosure is negative.

Sample size	Sample size was chosen based on sample availability as well as availability of funding for sequencing.
Data exclusions	Individuals inferred to be close (i.e., 1st degree) relatives were excluded. This was a pre-determined exclusion criterion, chosen because of genetic correlations between close relatives.
Replication	22 individuals were sequenced independently by ourselves and the 1000 Genomes Project. Non-reference discordance rates are presented in Supplementary Table 1. In all cases, the replication was successful (i.e., the discordance rates were extremely low).
Randomization	Not applicable. This was a descriptive study with no experimental groups.
Blinding	Not relevant, since there was no experimental manipulation. In addition, group membership was determined by sampling location, so blinding was impossible.

Reporting for specific materials, systems and methods

We require information from authors about some types of materials, experimental systems and methods used in many studies. Here, indicate whether each material, system or method listed is relevant to your study. If you are not sure if a list item applies to your research, read the appropriate section before selecting a response.

Materials & experimental systems

n/a	Involved in the study
☒	☐ Antibodies
☒	☐ Eukaryotic cell lines
☒	☐ Palaeontology and archaeology
☒	☐ Animals and other organisms
☒	☐ Clinical data
☒	☐ Dual use research of concern
☒	☐ Plants

Methods

n/a	Involved in the study
☒	☐ ChIP-seq
☒	☐ Flow cytometry
☒	☐ MRI-based neuroimaging
